# Impact of high fasting plasma glucose on liver cancer burden in China: a comprehensive analysis of trends from 1990 to 2021

**DOI:** 10.3389/fnut.2025.1628726

**Published:** 2025-09-09

**Authors:** Zhouwei Zhan, Wei Lin, Xintong Yao, Shuqi Huang, Shuangting Lan, Lina Zheng, Zengqing Guo, Bijuan Chen

**Affiliations:** ^1^Department of Medical Oncology, Clinical Oncology School of Fujian Medical University, Fujian Cancer Hospital, Fuzhou, Fujian, China; ^2^Clinical Oncology School of Fujian Medical University, Fujian Cancer Hospital, Fuzhou, Fujian, China; ^3^Department of Radiation Oncology, Clinical Oncology School of Fujian Medical University, Fujian Cancer Hospital, Fuzhou, Fujian, China

**Keywords:** liver cancer, high fasting plasma glucose, joinpoint regression analysis, age period-cohort analysis, burden, trends, China

## Abstract

**Background:**

High fasting plasma glucose (HFPG), a key metabolic risk factor, has emerged as a major contributor to the global cancer burden, particularly liver cancer. However, little is known about the long-term trends and sex-specific patterns of HFPG-attributable liver cancer in China.

**Methods:**

Data were obtained from the Global Burden of Disease Study 2021 to estimate the liver cancer burden attributable to HFPG in China from 1990 to 2021. Metrics analyzed included age-standardized mortality rates (ASMR), disability-adjusted life years (DALYs), years lived with disability (YLDs), and years of life lost (YLLs). Joinpoint regression and decomposition analyses were conducted to evaluate temporal trends and contributing factors.

**Results:**

In 2021, 3,467 deaths and 83,113 DALYs were attributable to HFPG-related liver cancer in China, with males bearing a higher burden than females. Mortality and DALYs peaked in individuals aged 65–69, and age-standardized rates rose with advancing age, especially among men. Between 1990 and 2021, the absolute burden increased, driven by population aging and growth, although age-standardized rates remained relatively stable. Joinpoint regression showed fluctuating trends with recent declines in DALYs and YLLs, especially in females. Compared to global trends, China exhibited more stability in age-standardized rates. Decomposition analysis identified aging as the primary driver of increased deaths, while population growth led the rise in DALYs. Epidemiological changes slightly offset DALYs in women but increased the burden in men.

**Conclusion:**

Despite stable age-standardized rates, the absolute burden of HFPG-related liver cancer in China has increased due to demographic shifts, with males and older adults disproportionately affected. Targeted interventions addressing metabolic risk factors in aging populations, particularly men, are urgently needed.

## Introduction

Liver cancer remains one of the most lethal malignancies worldwide, ranking among the top causes of cancer-related mortality. China alone accounts for over half of the global liver cancer deaths, largely due to its high burden of chronic hepatitis infections, rapid demographic transitions, and increasing prevalence of metabolic disorders (1–3). In recent years, metabolic dysfunction encompassing obesity, insulin resistance, and type 2 diabetes mellitus has been increasingly recognized as a major contributor to the development of hepatic carcinogenesis (4, 5). Among metabolic factors, high fasting plasma glucose (HFPG) has emerged as an independent and modifiable risk for liver cancer, contributing significantly to both incidence and mortality (6). As HFPG prevalence continues to climb in China amid an aging population and lifestyle shifts, its public health impact on liver cancer demands urgent evaluation (7–9). Understanding the specific burden of liver cancer attributable to HFPG is essential for guiding future cancer prevention and metabolic risk reduction strategies tailored to China’s epidemiological landscape.

Accumulating evidence from large-scale epidemiological studies supports a robust link between elevated blood glucose levels and primary liver cancer, independent of viral hepatitis or alcohol-related liver disease (10, 11). Prospective cohorts in both Eastern and Western populations have demonstrated a dose-dependent relationship between fasting glucose and liver cancer risk, with hyperglycemia accelerating hepatic inflammation, fibrosis, and malignant transformation (12). Biological mechanisms implicate chronic insulin resistance and hyperinsulinemia as key mediators of oncogenesis, partly through activation of insulin/IGF-1 signaling pathways and metabolic reprogramming (13, 14). Moreover, recent molecular studies reveal that high glucose levels can directly modulate tumor cell behavior by enhancing pro-tumorigenic pathways, such as Wnt/β-catenin signaling and O-GlcNAcylation-dependent transcription (15, 16). These findings underscore the multifaceted role of HFPG in liver tumorigenesis and suggest that mitigating glucose dysregulation may yield substantial benefit in reducing liver cancer burden.

Despite the growing recognition of HFPG as a risk factor, few studies have comprehensively quantified the long-term burden of liver cancer attributable to HFPG at the national level in China. Existing estimates tend to focus either on the general cancer burden or on diabetes-related liver disease without disaggregating the specific contribution of elevated fasting glucose (17, 18). Leveraging data from the Global Burden of Disease (GBD) Study 2021, this study aims to address this gap by analyzing national trends in deaths, disability-adjusted life years (DALYs), years lived with disability (YLDs), and years of life lost (YLLs) due to liver cancer attributable to HFPG in China from 1990 to 2021. By applying joinpoint regression, decomposition analysis, and age-period-cohort modeling, we provide a detailed evaluation of temporal changes, sex-specific disparities, and underlying demographic drivers. These insights are critical for identifying high-risk populations and informing metabolic disease prevention strategies. As the burden of glucose-related liver cancer shifts with age and population structure, our findings may also support broader policies integrating glycemic control into liver cancer prevention frameworks in China and other transitioning nations.

## Methods

### Data sources

Data on the burden of liver cancer attributable to HFPG in China from 1990 to 2021 were obtained from the GBD 2021, conducted by the Institute for Health Metrics and Evaluation (IHME) at the University of Washington. The GBD study provides comprehensive and comparable estimates of disease burden for 371 diseases and injuries, 88 risk factors, and 204 countries and territories (19, 20). To ensure data quality, all data sources underwent rigorous standardization and validation processes. For this study, we extracted data specific to China on liver cancer mortality, DALYs, YLDs, and YLLs attributable to HFPG. These data were stratified by sex, age group (in 5-year intervals from 25 to 29 to 95+), and year. Both all-age counts and age-standardized rates per 100,000 population were analyzed. HFPG-related burden estimates were derived from the comparative risk assessment (CRA) framework used in the GBD, which quantifies the disease burden attributable to specific risk factors by comparing observed health outcomes to those that would be expected under a theoretical minimum risk exposure level (19, 21). All estimates included 95% uncertainty intervals (UIs), calculated from the 2.5th and 97.5th percentiles of 1,000 draws from the posterior distribution, reflecting uncertainty from model inputs, sampling error, and measurement variability.

### Definition

In the GBD 2021, liver cancer is defined as malignant neoplasms of the liver, encompassing International Classification of Diseases, 10th Revision (ICD-10) codes C22.0-C22.8 and a proportion of C22.9 consistent with primary liver cancer. This definition primarily includes hepatocellular carcinoma (HCC), the most common histologic subtype globally. HFPG is defined as a fasting plasma glucose concentration exceeding the theoretical minimum risk exposure level (TMREL) of 4.9–5.3 mmol/L (88.2–95.4 mg/dL), based on meta-analytical evidence of minimal risk for chronic disease outcomes and all-cause mortality at this range (19, 20). The estimation of liver cancer burden attributable to HFPG was based on the CRA framework developed by the IHME. This method involved calculating the population-attributable fraction (PAF) using the distribution of HFPG exposure in the population and the relative risks of liver cancer across exposure levels, compared against the TMREL. The PAFs were then applied to liver cancer mortality and disability estimates to derive the HFPG-attributable burden. Estimates included the number and age-standardized rates (per 100,000 population) of deaths, DALYs, YLDs, and YLLs, disaggregated by age, sex, and calendar year. DALYs were calculated as the sum of YLLs and YLDs.

### Burden indicators estimation

The burden indicators, including DALYs, YLDs, and YLLs, along with the PAFs for liver cancer related to HFPG were calculated following the established GBD 2021 methodology (19, 20). Cause-specific mortality and YLLs were estimated using vital registration systems, cancer registries, and verbal autopsy data. YLDs were derived by multiplying the prevalence of each sequela (defined by ICD-10 codes C22.0-C22.8 and part of C22.9) by corresponding disability weights, estimated via population surveys. To model disease incidence, prevalence, and remission, the Bayesian meta-regression tool DisMod-MR 2.1 was employed. This approach uses a compartmental framework to ensure internal consistency among epidemiological parameters. Exposure levels for HFPG were estimated using a combination of large-scale national surveys and published datasets, synthesized with spatiotemporal Gaussian process regression (ST-GPR) models to generate continuous exposure surfaces by age, sex, location, and year. Theoretical minimum-risk exposure levels (TMREL, 4.9–5.3 mmol/L) and relative risks were obtained from meta-analyses of cohort studies, and these were used to calculate PAFs via comparative risk assessment (22). ASR (per 100,000 people) were calculated using the following formula: ASR = $\frac{\sum_{i=1}^{N}\mathrm{ai}*\mathrm{\omega i}}{\sum_{i=1}^{N}\mathrm{\omega i}}\times 100,000$ , where *ai* represents the age-specific rate in the *i* th age group, and *ωi* denotes the number (or weight) of individuals in the same age group in a standard population. DALYs were then computed as the sum of YLLs and YLDs attributable to HFPG. All estimates incorporated 95% UIs (2.5th-97.5th percentiles) derived from 1,000 model draws to reflect uncertainty in data inputs and model parameters (22).

### Descriptive analysis

A descriptive analysis was performed to assess the temporal and demographic patterns of liver cancer burden attributable to HFPG in China from 1990 to 2021. The analysis included estimates of both the all-age number and age-standardized rates (per 100,000 population) of deaths, DALYs, YLDs, and YLLs, stratified by sex and age group. Age-standardized rates were computed using the GBD global standard population to allow for consistent comparisons over time and between subgroups. Burden estimates for the year 2021 were first examined to describe the current distribution across age and sex. Age-specific counts and rates of deaths, DALYs, YLDs, and YLLs were plotted to identify peak burden ages and to assess sex disparities. To visualize long-term trends, annual estimates from 1990 to 2021 were graphed to evaluate changes over time in both absolute numbers and age-standardized rates. In addition, comparative analyses were conducted to place China’s trends within the global context. Age-standardized rates of HFPG-related liver cancer burden in China were compared to corresponding global values across the study period. These comparisons provided insights into how China’s epidemiological patterns align with or diverge from global trajectories.

### Joinpoint regression analysis

Joinpoint regression analysis was employed to assess temporal trends in age-standardized mortality, DALYs, YLDs, and YLLs rates due to liver cancer attributable to HFPG in China from 1990 to 2021. This method identifies statistically significant changes in trend (i.e., joinpoints) and calculates the annual percent change (APC) for each segment, as well as the average annual percent change (AAPC) for the overall period. Analyses were conducted for both sexes combined and stratified by sex to explore gender-specific trend differences. The Joinpoint Regression Program (version 5.2.0) developed by the US National Cancer Institute was used for modeling. The number of joinpoints was selected using the Monte Carlo permutation method, with a maximum of five joinpoints allowed, and a significance level of 0.05 (23–25). Log-linear models were fitted to the age-standardized rates to ensure constant variance, and APCs were considered statistically significant when the 95% confidence interval did not include zero. This approach enabled the detection of non-linear temporal patterns and the quantification of time periods with accelerating or decelerating trends in HFPG-attributable liver cancer burden. The use of joinpoint regression complements descriptive analyses by offering more precise insights into how the burden has evolved in response to demographic, epidemiological, and health system changes over time.

### Age-period-cohort (APC) analysis

To disentangle the temporal effects of age, calendar period, and birth cohort on the trends in liver cancer burden attributable to HFPG in China, an APC analysis was conducted. This approach allows for the assessment of how risk varies across different age groups, time periods, and generational cohorts while controlling for the linear dependency among these factors (26, 27). APC models were applied to age-standardized mortality and DALY rates stratified by sex, using data from 1990 to 2021. Age was grouped into 15 five-year intervals from 25–29 to 95+, and calendar periods were divided into 5-year intervals from 1992–1996 to 2017–2021. Birth cohorts were calculated by subtracting the midpoint of each age group from the midpoint of each calendar period. The intrinsic estimator (IE) method was used to address the identification problem arising from the exact linear relationship among age, period, and cohort. This method has been widely adopted in epidemiological research for its ability to generate unbiased and interpretable coefficients. The APC model fitting was performed using the “Epi” package (version 2.51) in R (version 4.3.1), and the residual deviations between models and Akaike Information Criterion (AIC) were compared to determine the optimal model.

### Decomposition analysis

To quantify the contributions of demographic and epidemiological factors to changes in the absolute burden of liver cancer attributable to HFPG in China from 1990 to 2021, a decomposition analysis was conducted. This method partitions the total change in the number of deaths and DALYs into three components: population growth, population aging, and epidemiological changes (28). First, the difference in absolute numbers of HFPG-attributable deaths and DALYs between 1990 and 2021 was calculated. Using the Das Gupta method, the contribution of each individual factor was estimated while keeping the other two variables constant. Population growth reflects changes in total population size, aging captures shifts in the population age structure, and epidemiological change accounts for differences in age-specific and sex-specific HFPG-attributable liver cancer rates. These epidemiological changes reflect shifts in disease burden driven by evolving exposures to metabolic risk factors, improvements or deteriorations in public health infrastructure, changes in disease screening and diagnosis, and advancements in treatment strategies. This analysis was performed for the total population and stratified by sex to evaluate gender-specific drivers of change. The decomposition results provide insights into the relative influence of demographic dynamics versus temporal changes in population health and healthcare delivery. All decomposition calculations were performed using R software (version 4.3.1), and estimates were visually presented using bar plots to highlight the proportional contributions of each component over time.

## Results

### Burden of liver cancer attributable to HFPG in China, 2021

In 2021, the burden of liver cancer attributable to HFPG in China remained substantial, with a notable sex disparity. A total of 3,467 deaths were estimated to be linked to HFPG, with a higher burden observed in males than females. This trend was consistently reflected across other health loss metrics. The total DALYs attributable to HFPG reached 83,113, with males accounting for a greater proportion of this burden than females. YLLs made up the vast majority of DALYs, underscoring the fatal nature of HFPG-related liver cancer, whereas YLDs contributed minimally. When standardized for age, the burden remained higher in men than in women, with age-standardized DALY and YLL rates per 100,000 population in males approaching 4.5 and 4.46, respectively, compared to 3.25 and 3.21 in females (Table 1).

**Table 1:** All-age cases and age-standardized deaths, DALYs, YLDs, and YLLs rates in 2021 for liver cancer attributable to HFPG in China.

Measure	All-ages cases (95% UIs)			Age-standardized rates per 100,000 people (95% UIs)		
	Total	Male	Female	Total	Male	Female
Deaths	3,467 (376, 7,167)	1839 (201, 3,962)	1,628 (168, 3,409)	0.17 (0.02, 0.34)	0.19 (0.02, 0.41)	0.15 (0.02, 0.31)
DALYs	83,113 (9,107, 173,016)	46,764 (5,173, 100,851)	36,349 (3,798, 77,331)	3.86 (0.42, 8.04)	4.5 (0.49, 9.69)	3.25 (0.34, 6.94)
YLDs	886 (85, 1931)	488 (49, 1,105)	398 (38, 850)	0.04 (0, 0.09)	0.05 (0, 0.11)	0.04 (0, 0.08)
YLLs	82,227 (9,023, 171,100)	46,276 (5,116, 99,705)	35,951 (3,760, 76,564)	3.82 (0.42, 7.95)	4.46 (0.49, 9.57)	3.21 (0.33, 6.88)

Values in parentheses indicate 95% UIs, estimated using Monte Carlo simulations. DALYs, disability-adjusted life-years; YLDs, years lived with disability; YLLs, years of life lost; HFPG, high fasting plasma glucose; UIs, uncertainty intervals.

### Age and sex distribution of liver cancer burden attributable to HFPG in China, 2021

**Figure 1:**
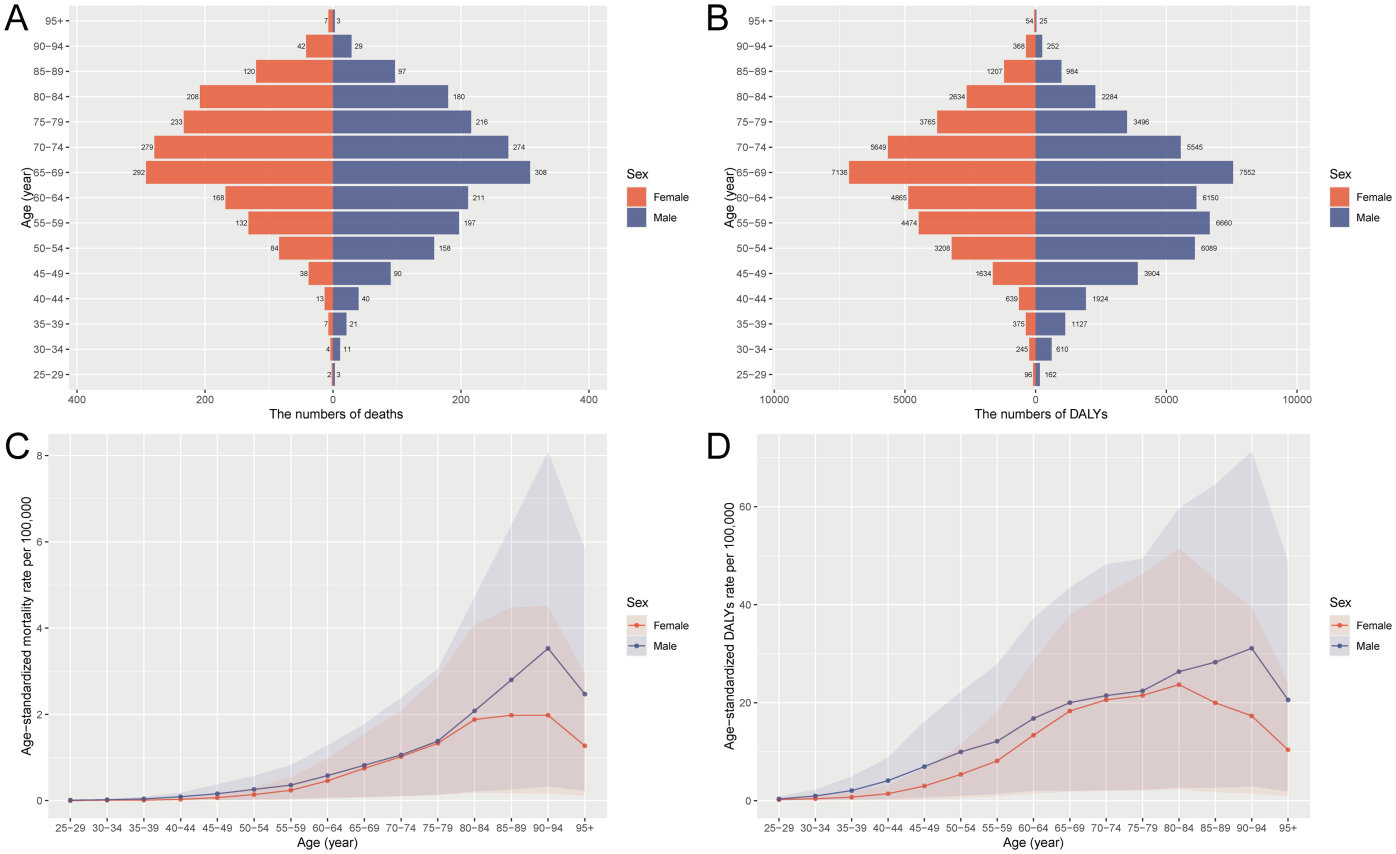
Number and age-standardized rates of deaths and DALYs due to liver cancer attributable to HFPG in China by age group and sex in 2021. **(A)** Number of deaths. **(B)** Number of DALYs. **(C)** Age-standardized mortality rate. **(D)** Age-standardized DALY rate. HFPG, high fasting plasma glucose; DALYs, disability-adjusted life years; ASMR, age-standardized mortality rate.

In 2021, the burden of liver cancer attributable to HFPG in China demonstrated pronounced variations by age and sex. The number of deaths rose sharply in older age groups, peaking at 65–69 years for both sexes, with males showing higher death counts across all age groups. DALYs followed a similar trend, increasing significantly with age and reaching their highest levels in the same age group, again with males bearing a greater burden. YLDs peaked at ages 65–69 in both sexes, while YLLs peaked at 55–59 and 65–69 years in males and at 65–69 and 70–74 years in females, underscoring the impact of premature mortality in these groups. When age-standardized rates were examined, all indicators (deaths, DALYs, YLDs, and YLLs) exhibited steep increases with age, particularly peaking around 85–89 years for both sexes. Across all measures, males consistently experienced higher rates than females, indicating a heavier burden of HFPG-related liver cancer among men (Figure 1 and Supplementary Figure S1).

### Trends over time for liver cancer burden attributable to HFPG in China, 1990–2021

**Figure 2:**
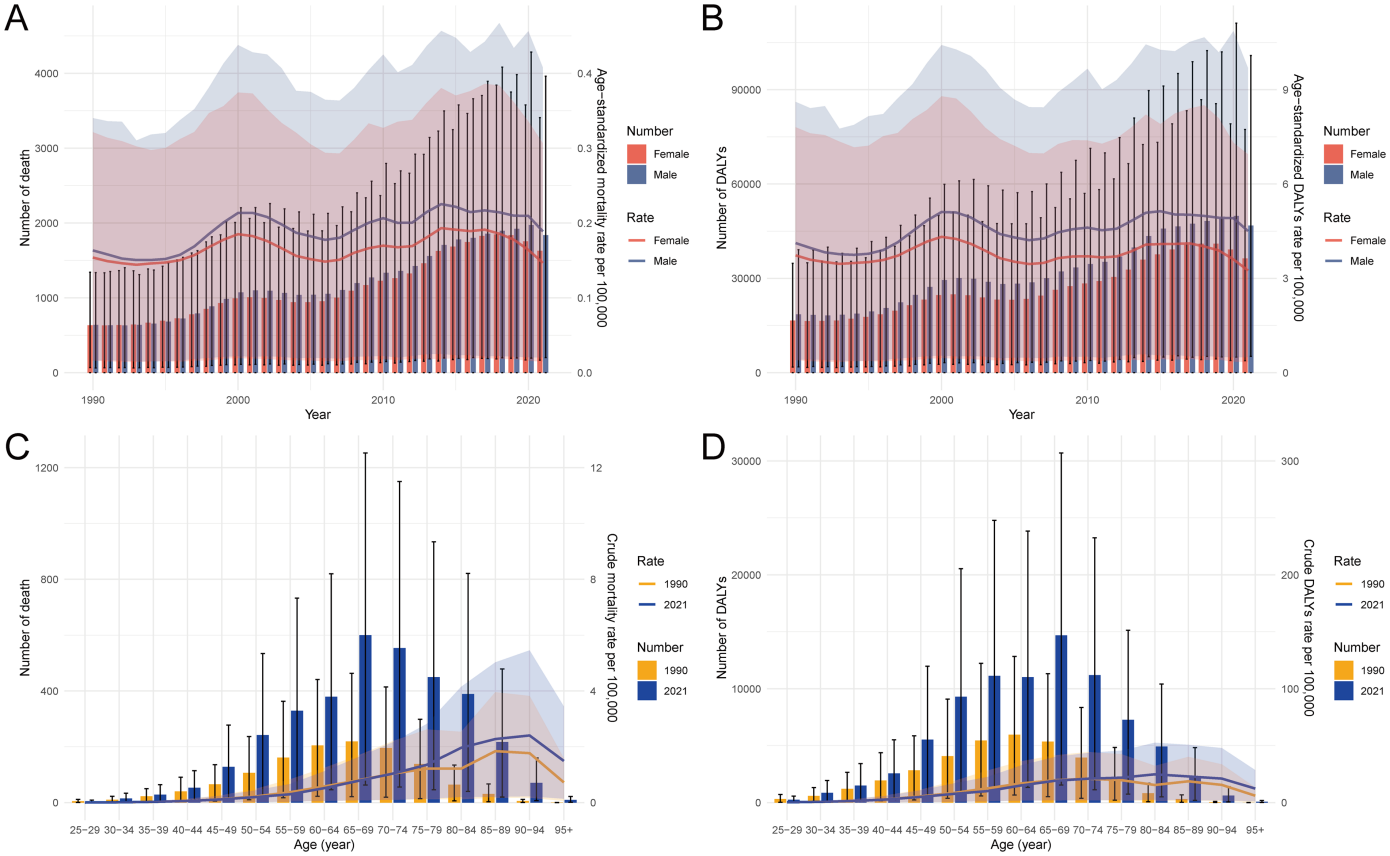
Temporal trends and age-specific distribution of deaths and DALYs due to liver cancer attributable to HFPG in China, 1990–2021. **(A)** Number and age-standardized rates of deaths by sex over time. **(B)** Number and age-standardized rates of DALYs by sex over time. **(C)** Number of deaths and crude death rates per 100,000 by age group in 1990 and 2021. **(D)** Number of DALYs and crude DALYs rates per 100,000 by age group in 1990 and 2021. HFPG, high fasting plasma glucose; DALYs, disability-adjusted life years.

Between 1990 and 2021, the number of deaths, DALYs, YLDs, and YLLs attributable to HFPG in China shows an overall fluctuating increase from 1990 to 2021, with observable variations at different time points. This upward trend in absolute numbers is more pronounced among males, who consistently exhibit a higher burden across all indicators. In contrast, the age-standardized rates of deaths, DALYs, YLDs, and YLLs have remained relatively stable throughout the period, suggesting that population growth and aging have primarily driven the increase in total burden. Notably, a slight downward trend in age-standardized rates has emerged in recent years across all four measures, indicating potential improvements in risk factor management or early intervention (Figure 2 and Supplementary Figures S2A,B).

### Age-specific trends in liver cancer burden attributable to HFPG in China, 1990–2021

From 1990 to 2021, older populations have consistently experienced a heavier burden of liver cancer attributable to HFPG in China, with the increase most prominent among individuals aged 75 years and above. As illustrated in Figure 2 and Supplementary Figure S2, not only have mortality rates risen substantially in these age groups, but YLDs and overall disease impact have also expanded, underscoring a growing burden in the aging demographic. While HFPG-related liver cancer remains present across all age groups, the trend indicates a shifting concentration of burden toward older adults. This transition is particularly evident in the sharp rise in crude death rates and YLLs among the elderly, reflecting an increasing role of premature mortality in driving the disease burden (Figure 2 and Supplementary Figures S2C,D).

### Comparison of age-standardized trends in China and globally, 1990–2021

Between 1990 and 2021, age-standardized rates of liver cancer attributable to HFPG remained relatively stable in China but increased significantly at the global level. As shown in Table 2 and Supplementary Figure S3, the ASMR, DALYs, YLDs, and YLLs rates in China showed only marginal changes over the three decades, with minor fluctuations and no statistically significant trends. For instance, the ASMR in China increased slightly from 0.16 to 0.17 per 100,000, while DALYs and YLLs rates decreased marginally. In contrast, global rates for all four indicators rose significantly, with statistically meaningful increases, including a 59% increase in ASMR and a 1.81-unit rise in age-standardized DALY rates. The most prominent global increase was seen in YLDs, which more than doubled. These findings suggest that although the absolute burden in China has grown, the age-specific risk has remained relatively steady, unlike the global context where HFPG-related liver cancer risk has escalated across populations.

**Table 2:** Change of age-standardized rates in deaths, DALYs, YLDs, and YLLs for liver cancer attributable to HFPG between 1990 and 2021 in China and global level.

Measure	China			Global		
	1990	2021	Change	1990	2021	Change
Deaths	0.16 (0.02, 0.33)	0.17 (0.02, 0.34)	0.23 (−0.47–0.94)	0.1 (0.01, 0.2)	0.17 (0.02, 0.33)	1.59 (1.44–1.74) ^*^
DALYs	3.93 (0.41, 8.04)	3.86 (0.42, 8.04)	0.09 (−0.39–0.57)	2.28 (0.24, 4.65)	3.67 (0.41, 7.36)	1.81 (1.66–1.95) ^*^
YLDs	0.03 (0, 0.07)	0.04 (0, 0.09)	0.77 (0.59–0.95)	0.02 (0, 0.05)	0.04 (0, 0.09)	2.12 (2.06–2.18) ^*^
YLLs	3.9 (0.41, 7.97)	3.82 (0.42, 7.95)	0.08 (−0.40–0.56)	2.26 (0.24, 4.6)	3.63 (0.41, 7.28)	1.58 (1.44–1.73) ^*^

Values in parentheses indicate 95% UIs, estimated using Monte Carlo simulations. Abbreviations: DALYs, disability-adjusted life-years; YLDs, years lived with disability; YLLs, years of life lost; HFPG, high fasting plasma glucose; UIs, uncertainty intervals; ^*^p < 0.05 (permutation test).

### Joinpoint regression analysis of trends in liver cancer burden attributable to HFPG in China, 1990–2021

**Figure 3:**
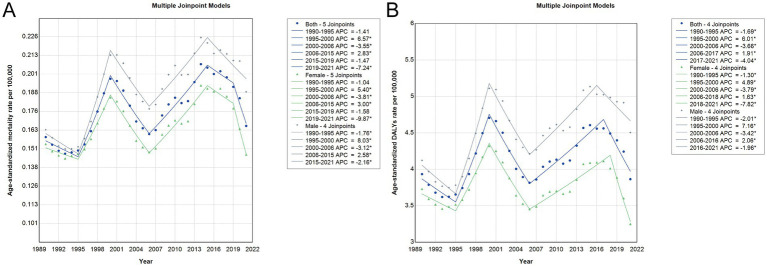
Joinpoint analysis of trends in age-standardized burden rates of liver cancer attributable to HFPG in China from 1990 to 2021. **(A)** ASMR. **(B)** DALYs rate. The analysis includes trends for both sexes combined (blue line), females (green line), and males (grey line), with asterisks indicating statistically significant changes (*p* < 0.05). ASMR, age-standardized mortality rates; DALYs, disability-adjusted life years; HFPG, high fasting plasma glucose.

Joinpoint regression analysis revealed distinct temporal patterns in age-standardized rates of liver cancer attributable to HFPG in China from 1990 to 2021, with notable differences by sex and indicator type. As shown in Figure 3, Supplementary Figure S4 and Supplementary Table S1, the trends were characterized by multiple phases of significant increases and decreases. Among both sexes, a sharp rise in all indicators occurred between 1995 and 2000, followed by a significant decline during the early 2000s, and a subsequent increase from the mid-2000s to mid-2010s. In recent years, a downward trend has emerged, with statistically significant declines observed from 2017 onward in DALYs and YLLs, particularly in females (AAPC = −7.82%) and to a lesser extent in males. Notably, while age-standardized YLD rates exhibited modest increases over the entire period (AAPC = 0.77% for both sexes), mortality and YLL rates remained largely stable in the long term, with average annual percent changes close to zero.

### Age, period, and cohort effects on liver cancer deaths and DALYs attributable to HFPG in China, 1990–2021

**Figure 4:**
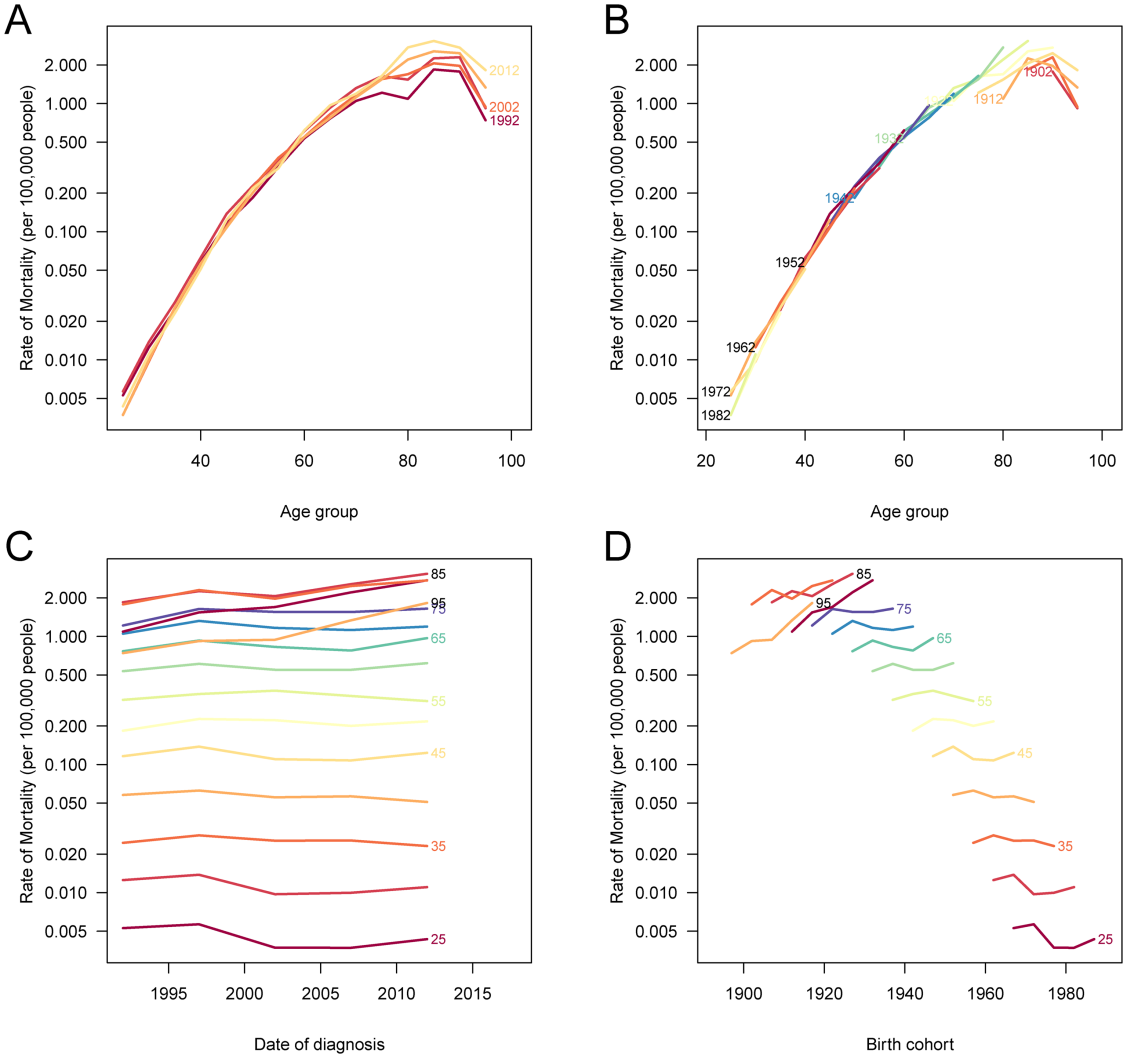
Age, period, and cohort effects on liver cancer deaths attributable to HFPG in China. **(A)** ASMR according to different time periods; each line connects the age-specific death rates for a 5-year period. **(B)** The period-specific death rates according to age groups; each line connects the period-specific death rates for a 5-year age group. **(C)** The cohort-specific death rates according to age groups; each line connects the cohort-specific death rates for a 5-year birth cohort. **(D)** The cohort-specific death rates according to different periods; each line connects the cohort-specific death rates for a 5-year period. HFPG, high fasting plasma glucose; ASMR, age-standardized mortality rates.

Age-specific patterns show that both mortality and DALYs increase with advancing age, indicating a heavier burden in older populations. Period-specific analyses reveal variations across different time periods, reflecting the influence of societal changes, healthcare advancements, and shifts in HFPG prevalence over time. The cohort effects underscore that more recent birth cohorts experience higher rates of mortality and DALYs as they age compared to earlier cohorts, suggesting a rising vulnerability in younger generations as they reach older age. The analysis of birth cohort-specific trends across different periods further emphasizes that individuals born in more recent cohorts face a greater burden as they age, highlighting the long-term impact of changing metabolic risks (Figure 4 and Supplementary Figure S5).

### Decomposition of changes in liver cancer deaths and DALYs attributable to HFPG in China, 1990–2021

**Figure 5:**
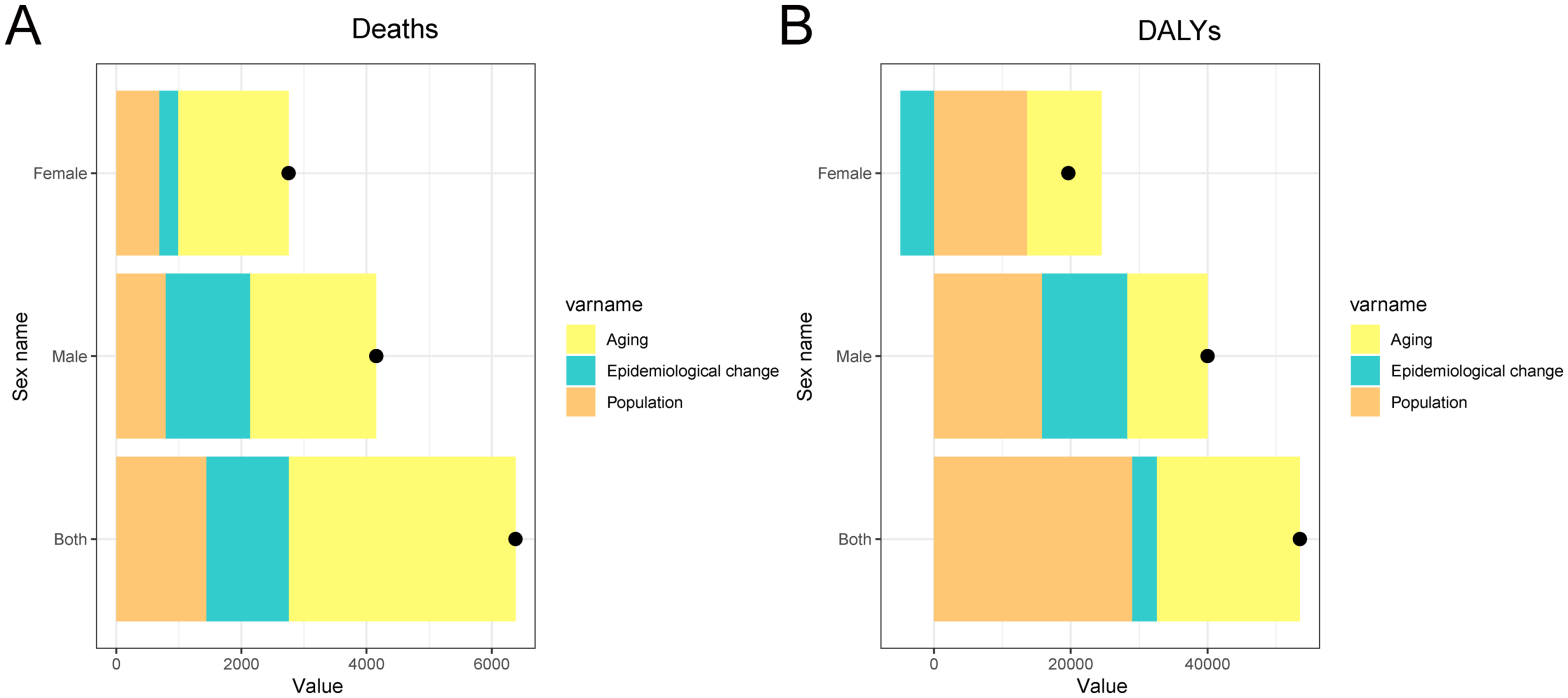
Decomposition of changes in the number of deaths **(A)** and DALYs **(B)** due to liver cancer attributable to HFPG in China by sex from 1990 to 2021. DALYs, disability-adjusted life years; HFPG, high fasting plasma glucose.

As shown in Figure 5, decomposition analysis revealed distinct patterns in the driving forces behind changes in HFPG-related liver cancer burden in China from 1990 to 2021, with notable gender differences. For deaths, aging emerged as the most significant contributor, followed by epidemiological changes and population growth, with all three factors exerting a more pronounced influence among men. In contrast, the decomposition of DALYs showed that population growth was the leading driver, followed by aging and then epidemiological changes. Interestingly, while all three components acted as positive drivers among men, epidemiological change served as a protective factor for women, mitigating their overall DALY burden.

## Discussion

This study provides a comprehensive assessment of the burden of liver cancer attributable to HFPG in China from 1990 to 2021. Our findings reveal that in 2021 alone, HFPG was associated with over 3,400 liver cancer deaths and more than 83,000 DALYs, with males experiencing consistently higher burden across all metrics. The age distribution of burden demonstrated a marked concentration in older age groups, particularly those aged 65–69 years, with disease burden rising steeply with age. Temporal analyses showed that while the absolute number of deaths, DALYs, YLDs, and YLLs increased steadily over the past three decades, age-standardized rates remained largely stable, with a slight downward trend emerging in recent years. These patterns indicate that the rising burden is primarily driven by demographic factors such as aging and population growth rather than a substantial shift in age-specific risk. Joinpoint regression highlighted significant fluctuations over time, including a marked increase in burden between 1995 and 2000, followed by a decline and subsequent resurgence in the mid-2000s. Decomposition analysis confirmed that aging was the principal contributor to the increase in HFPG-related liver cancer deaths, while population growth accounted for most of the rise in DALYs. Notably, sex-specific analyses showed that men consistently bore a higher burden, and epidemiological changes had a protective effect on women’s DALYs. These findings underscore the evolving demographic and metabolic landscape of liver cancer in China and highlight the urgency of integrating glycemic control into national cancer prevention strategies.

Mechanistically, liver cancer attributable to HFPG arises from a complex interplay of metabolic disruptions triggered by chronic hyperglycemia. Sustained elevations in fasting glucose promote hepatic steatosis, inflammation, and insulin resistance, which together create a pro-oncogenic hepatic environment (29, 30). Hyperinsulinemia, a characteristic feature of insulin resistance, stimulates both the insulin and insulin-like growth factor-1 (IGF-1) signaling pathways, which are closely associated with the development of HCC by promoting mitogenic activity, suppressing apoptosis, and disrupting normal cell proliferation (31). IGF-1, in particular, accelerates cell cycle progression while preventing programmed cell death, facilitating tumor progression (32, 33). Additionally, insulin has been shown to upregulate Bcl-xl expression and promote hepatoma cell migration and invasiveness, further exacerbating malignancy (34). These biological mechanisms are consistent with findings from multiple population-based studies and meta-analyses, which consistently identify HFPG and type 2 diabetes as significant independent risk factors for liver cancer. A recent umbrella review of prospective cohorts reported the strongest association between impaired fasting glucose and liver cancer among several obesity-and glucose-related malignancies (35). Hazard ratios of 1.54 in Korean populations (36) and up to 1.78 in Chinese populations confirm a robust epidemiological relationship (37–39). Importantly, the risk appears to be elevated in individuals with early-onset or long-standing diabetes, suggesting that glycemic duration may be an important modifier of cancer risk. These mechanistic and epidemiologic findings reinforce the need to integrate glycemic control into broader cancer prevention strategies, particularly for populations with sustained hyperglycemia.

Our findings align with previous studies indicating that males are more susceptible to liver cancer attributable to HFPG, a pattern observed globally (17). This disparity may be due to the higher prevalence and faster increase in glycemic disorders among men, compounded by other common liver cancer risk factors such as alcohol consumption and metabolic syndrome, which are also more prevalent in males (40). These overlapping risks may synergistically enhance the oncogenic potential of HFPG and lead to poorer outcomes. To address this sex-specific burden, comprehensive prevention strategies tailored to men are essential, including enhanced ultrasonographic liver cancer screening and structured follow-up programs. Notably, our findings suggest a narrowing of gender disparities in liver cancer burden after menopause, possibly driven by estrogen decline and reduced metabolic protection in postmenopausal women (41, 42). Decomposition analysis identified population aging as the leading driver of increased mortality, underscoring the substantial impact of demographic aging on disease trends in China (43). As global rates of obesity and diabetes continue to climb alongside aging populations, a further rise in liver cancer incidence is expected (44). Additionally, population growth significantly contributed to the rising DALY burden, with gender-specific disparities that may stem from differences in healthcare access and exposure to risk factors. Interestingly, while global age-standardized incidence rates of HFPG-related liver cancer have risen, China’s trend remained stable, likely due to longstanding HBV control programs and chronic disease management policies that may have mitigated the impact of rising metabolic risks. Conversely, in many developing regions, the absence of such systematic interventions, coupled with increasing obesity and diabetes prevalence, may have contributed to the sharp global rise (45). APC analysis showed that more recent birth cohorts are at higher mortality risk, indicating a shift in disease burden toward younger populations. This trend highlights the urgency of implementing early intervention strategies targeting younger individuals, including lifestyle modification and metabolic risk control (46).

The findings of this study have important clinical and public health policy implications for liver cancer prevention in China. Given the strong and consistent association between HFPG and liver cancer risk, integrating metabolic risk screening into routine clinical practice is essential. Targeted interventions should prioritize individuals with impaired glucose metabolism, particularly those with long-standing diabetes or early-onset disease, who appear to carry the highest risk (47). Regular liver ultrasonography for high-risk groups (48), coupled with systematic monitoring of glycemic control, may facilitate earlier detection and improve outcomes. At the population level, public health strategies should focus on reducing modifiable metabolic risks through comprehensive programs that promote healthy diet, physical activity, weight management, and diabetes education. These initiatives are particularly urgent in light of the growing prevalence of obesity and diabetes in younger cohorts, as identified in our birth cohort analysis. From a policy perspective, addressing the gender disparity in burden requires improving access to metabolic and cancer care services for men, who are disproportionately affected. At the same time, greater attention should be paid to women in the postmenopausal stage, as their relative risk increases with hormonal changes. Health system preparedness is also critical, especially as population aging continues to drive up absolute burden. Resource allocation and service delivery planning must account for this demographic shift by scaling up liver cancer screening, primary care management of metabolic disease, and integration of non-communicable disease control into cancer prevention frameworks.

Despite the strengths of this study, including the use of standardized, comprehensive estimates from the GBD 2021 and advanced analytical methods, several limitations should be acknowledged. First, the estimates of liver cancer burden attributable to HFPG rely on modeled data and assumptions inherent in the comparative risk assessment framework, which may introduce uncertainty in attribution, particularly when primary data are sparse in some regions or subpopulations (49, 50). Second, although the study adjusts for key covariates, potential residual confounding cannot be fully ruled out. For example, hepatitis B virus (HBV) infection, a major risk factor for liver cancer in China, may interact with metabolic risk factors, yet synergistic effects between HFPG and HBV are not explicitly modeled. Third, HFPG exposure was assessed at the population level rather than individual level, which may limit the precision of burden attribution in certain subgroups. Additionally, this study did not evaluate the effects of glucose-lowering medications, which have been shown to influence cancer outcomes and may confound the observed associations (51). Future research should aim to incorporate individual-level cohort data, including glycemic history, comorbidities, medication use, and HBV status, to refine risk estimates and better understand causal pathways. Moreover, further investigation is needed to identify the effectiveness of targeted interventions, particularly among high-risk groups such as younger individuals with early metabolic dysregulation and older adults with multiple comorbidities, to inform clinical practice and guide public health resource allocation.

## Conclusion

This study reinforces the growing importance of metabolic factors in shaping the landscape of liver cancer burden in China. The integration of high fasting plasma glucose as a key risk factor into national cancer prevention and metabolic disease control strategies is both timely and essential. As demographic shifts continue to accelerate, and as lifestyle transitions increasingly affect younger populations, the intersection of glycemic disorders and cancer warrants heightened clinical and policy attention. Future research should focus on elucidating individual-level interactions between glycemic control, comorbid conditions, and liver cancer risk, as well as evaluating the long-term effectiveness of targeted interventions in diverse population groups. Emphasis should also be placed on generating region-specific data, particularly for underserved areas, to improve risk stratification and intervention precision. A more refined understanding of metabolic-cancer linkages will be vital in supporting effective, equitable, and sustainable cancer prevention frameworks in the era of chronic disease convergence.

## Data Availability

Publicly available datasets were analyzed in this study. This data can be found: https://vizhub.healthdata.org/gbd-results/.

## Supplementary material

SUPPLEMENTARY FIGURE S1 Number and age-standardized rates of YLDs and YLLs due to liver cancer attributable to HFPG in China by age group and sex in 2021. **(A)** Number of YLDs. **(B)** Number of YLLs. **(C)** Age-standardized YLD rate. **(D)** Age-standardized YLL rate. HFPG, high fasting plasma glucose; YLDs, years lived with disability; YLLs, years of life lost.

SUPPLEMENTARY FIGURE S2 Temporal trends and age-specific distribution of YLDs and YLLs due to liver cancer attributable to HFPG in China, 1990–2021. **(A)** Number and age-standardized rates of YLDs by sex over time. **(B)** Number and age-standardized rates of YLLs by sex over time. **(C)** Number of YLDs and crude YLD rates per 100,000 by age group in 1990 and 2021. **(D)** Number of YLLs and crude YLL rates per 100,000 by age group in 1990 and 2021. HFPG, high fasting plasma glucose; YLDs, years lived with disability; YLLs, years of life lost.

SUPPLEMENTARY FIGURE S3 Trends in global and China-specific age-standardized rates of liver cancer attributable to HFPG from 1990 to 2021. **(A)** Global trends. **(B)** Trends in China. The figure illustrates the ASMR, DALYs rate, YLDs rate, and YLLs rate per 100,000 population over time. HFPG, high fasting plasma glucose; ASMR, age-standardized mortality rates; DALYs, disability-adjusted life years; YLDs, years lived with disability; YLLs, years of life lost.

SUPPLEMENTARY FIGURE S4 Joinpoint analysis of trends in age-standardized burden rates of liver cancer attributable to HFPG in China from 1990 to 2021. **(A)** YLDs rate. **(B)** YLLs rate. The analysis includes trends for both sexes combined (blue line), females (green line), and males (grey line), with asterisks indicating statistically significant changes (*p* < 0.05). YLDs, years lived with disability; YLLs, years of life lost; HFPG, high fasting plasma glucose.

SUPPLEMENTARY FIGURE S5 Age, period, and cohort effects on liver cancer DALYs attributable to HFPG in China. **(A)** Age-specific DALYs across different time periods, showing how DALYs shift across age groups from 1990 to 2021. **(B)** Period-specific DALYs across different age groups, illustrating changes in the burden over various time periods. **(C)** Cohort-specific DALYs, highlighting trends among different birth cohorts, showing how the liver cancer burden has evolved across generations. **(D)** Period-specific DALY rates across different birth cohorts, illustrating shifts in the burden over time and cohort effects on DALYs associated with HFPG-related liver cancer. HFPG, high fasting plasma glucose; DALYs, disability-adjusted life years.

## Glossary

HFPG: high fasting plasma glucose
GBD: Global Burden of Disease
ASMR: age-standardized mortality rates
DALYs: disability-adjusted life years
YLDs: years lived with disability
YLLs: years of life lost
IHME: Institute for Health Metrics and Evaluation
ICD: International Classification of Diseases
HCC: hepatocellular carcinoma
CRA: comparative risk assessment
UIs: uncertainty intervals
TMREL: theoretical minimum risk exposure level
PAF: population-attributable fraction
APC: annual percent change
AAPC: average annual percent change
IE: intrinsic estimator
APC: age-period-cohort
AIC: Akaike Information Criterion
HBV: hepatitis B virus
IGF-1: insulin-like growth factor-1
