# Supplementary figures and images for: Impact of high fasting plasma glucose on liver cancer burden in China: a comprehensive analysis of trends from 1990 to 2021
Source: Front Nutr. 2025 Sep 9;12:1628726. doi: 10.3389/fnut.2025.1628726 (PMC12454373; doi:10.3389/fnut.2025.1628726)

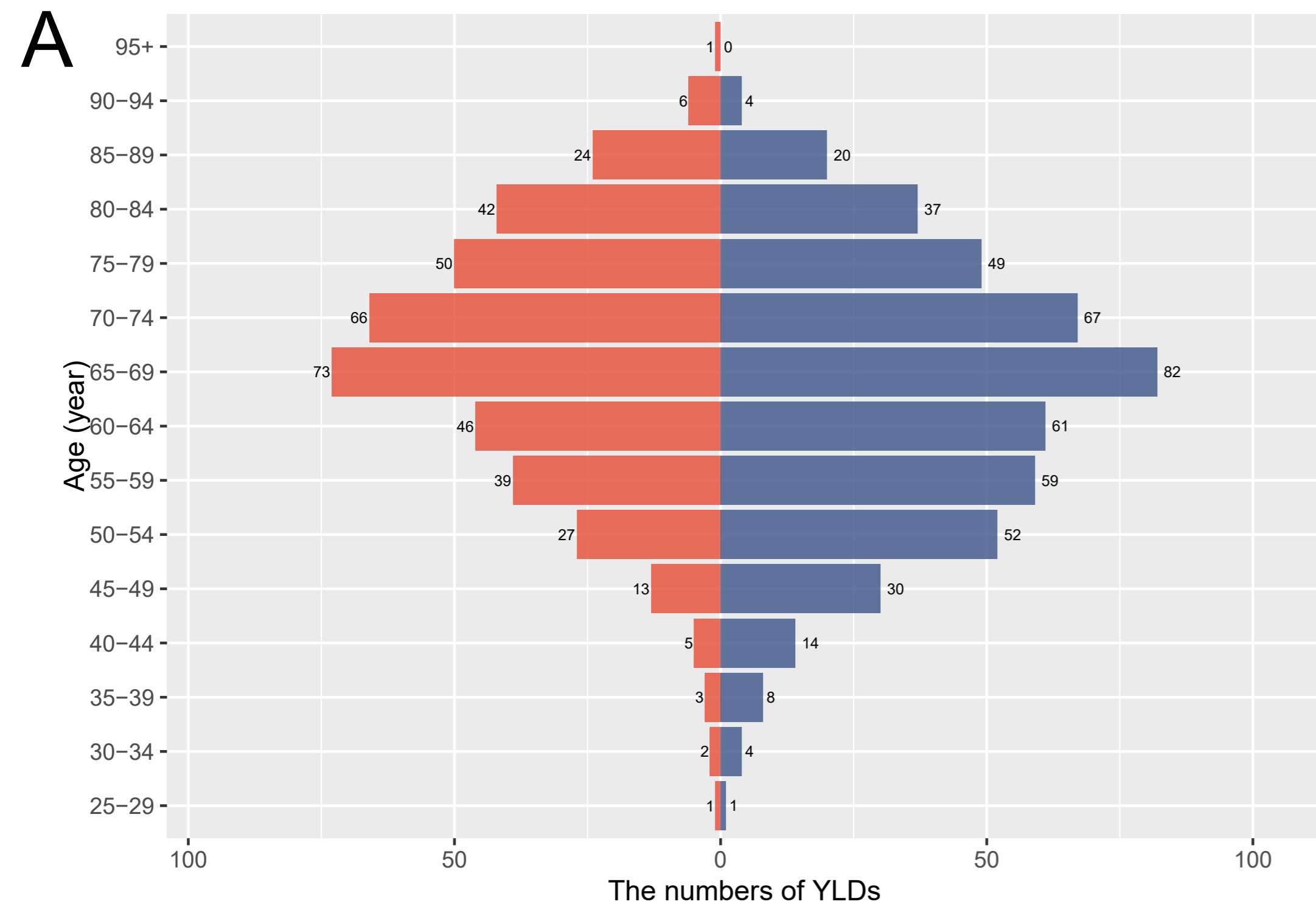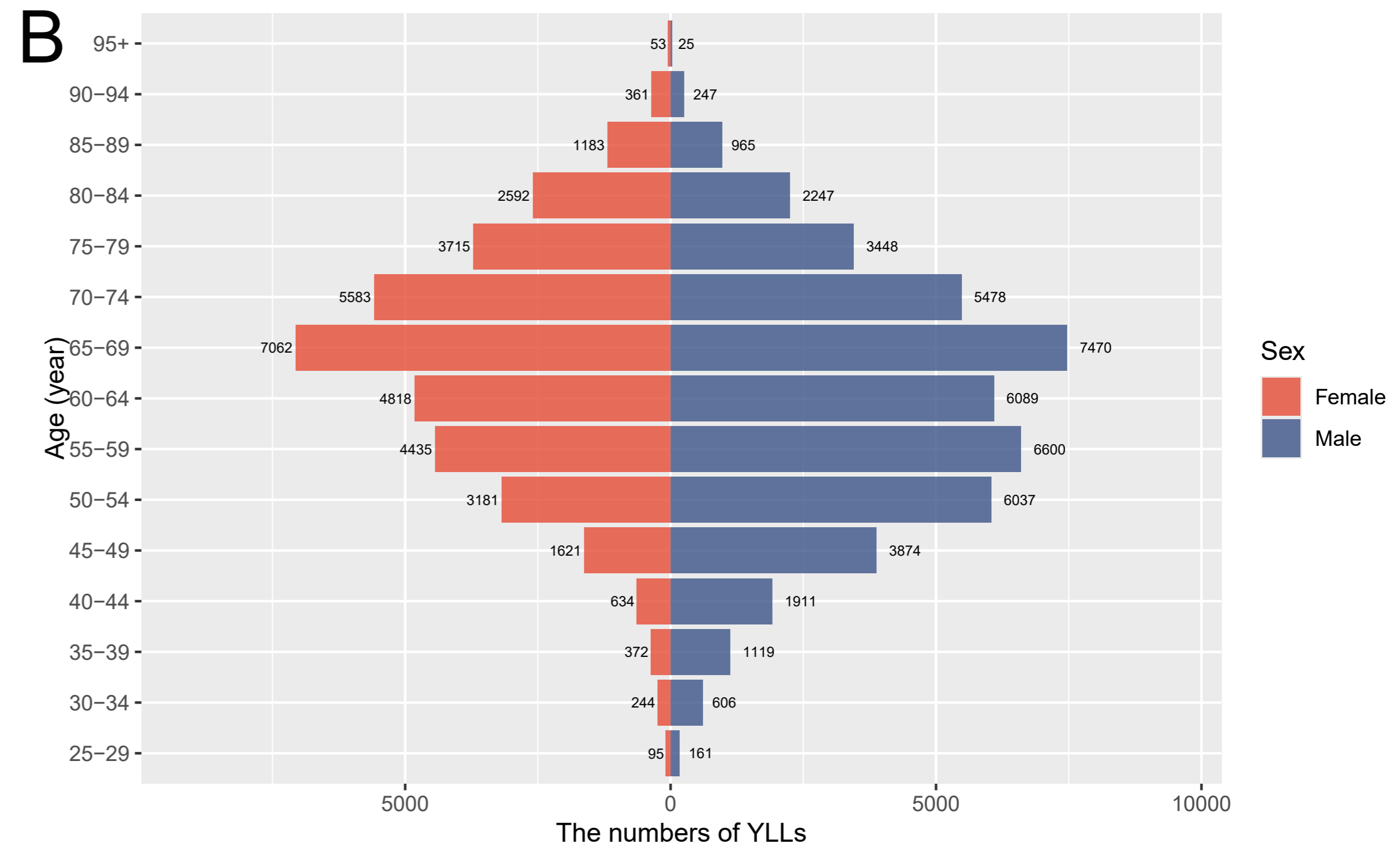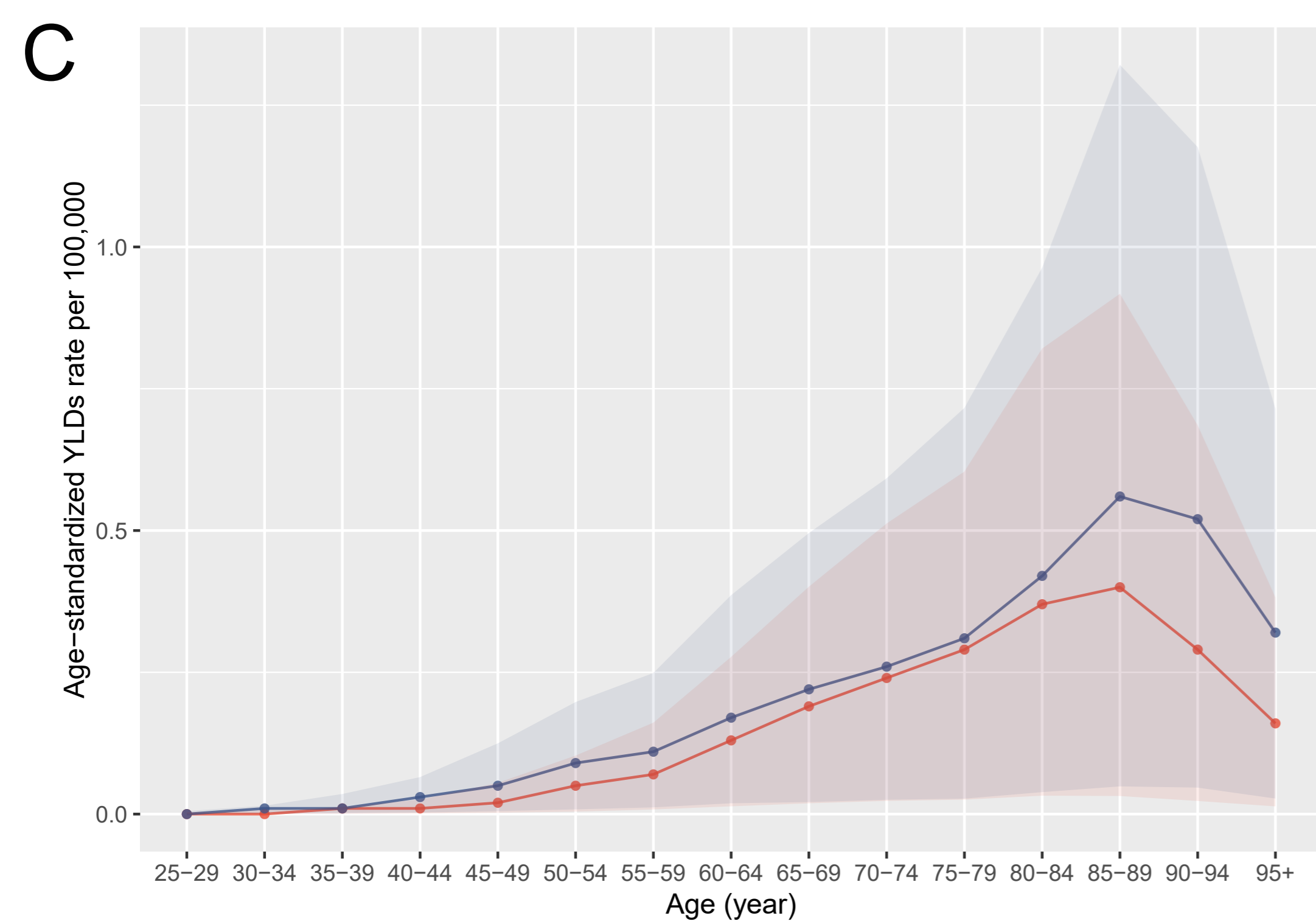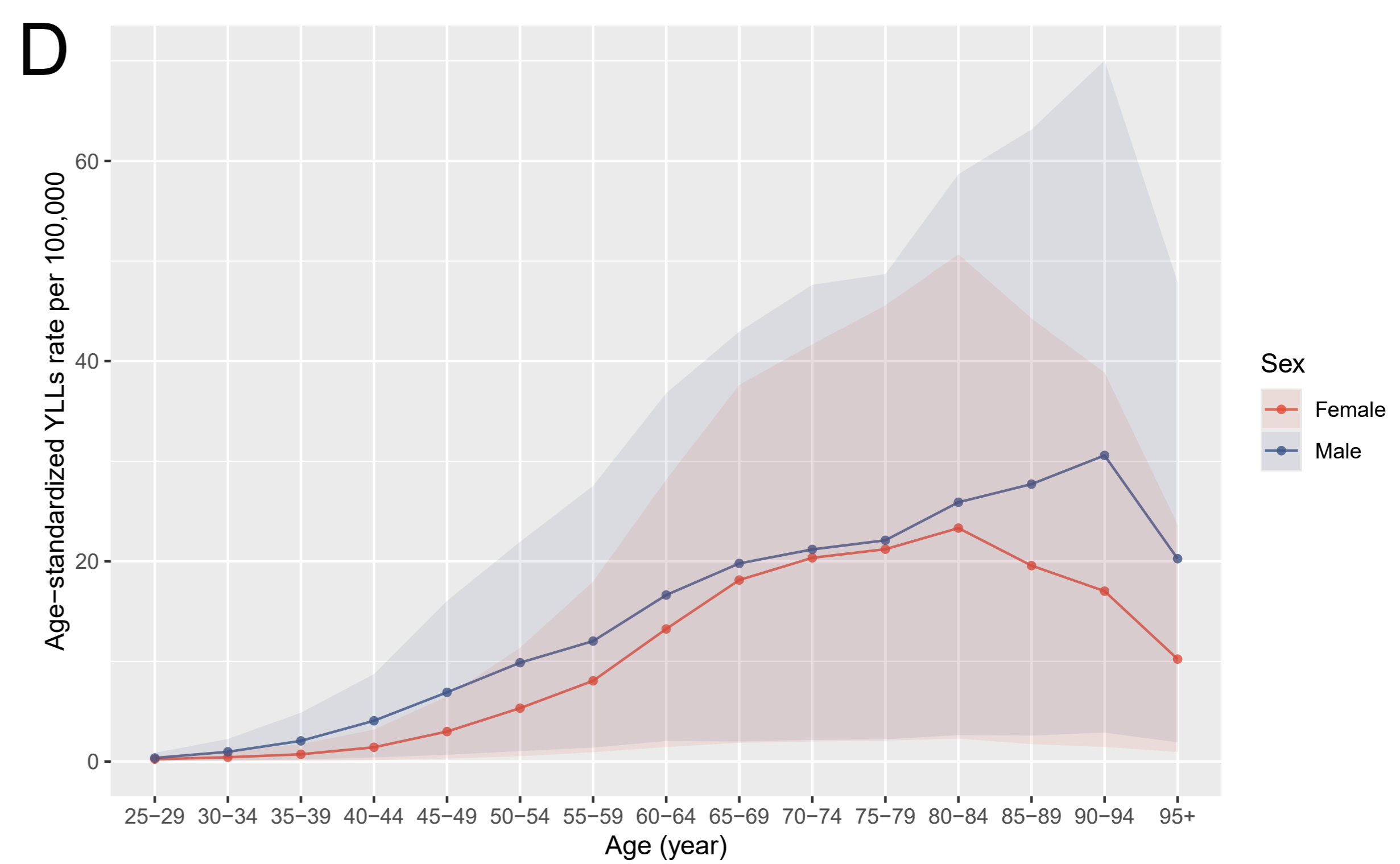

Supplement: SUPPLEMENTARY FIGURE S1 — Number and age-standardized rates of YLDs and YLLs due to liver cancer attributable to HFPG in China by age group and sex in 2021. (A) Number of YLDs. (B) Number of YLLs. (C) Age-standardized YLD rate. (D) Age-standardized YLL rate. HFPG, high fasting plasma glucose; YLDs, years lived with disability; YLLs, years of life lost. [file Image_1.pdf]

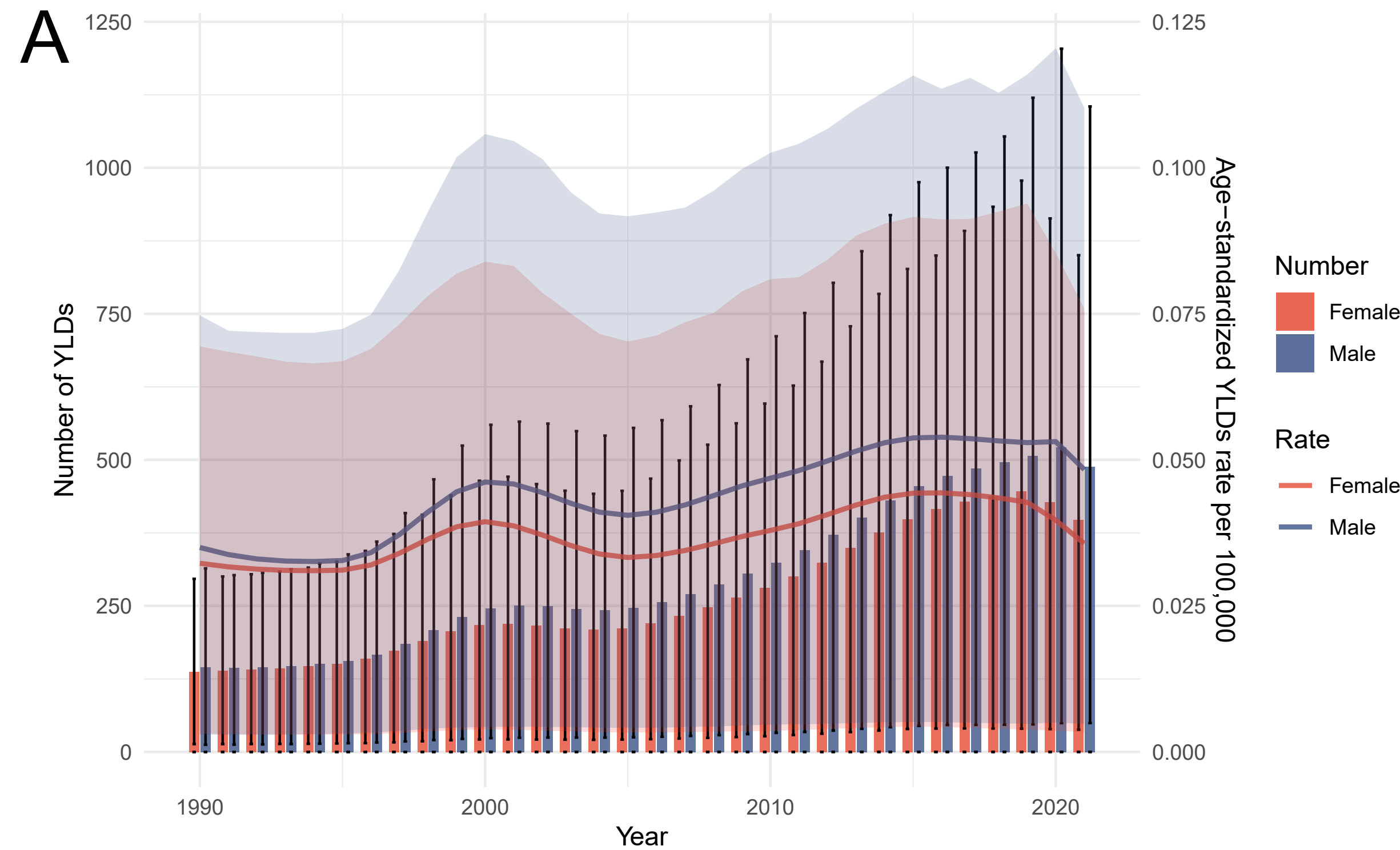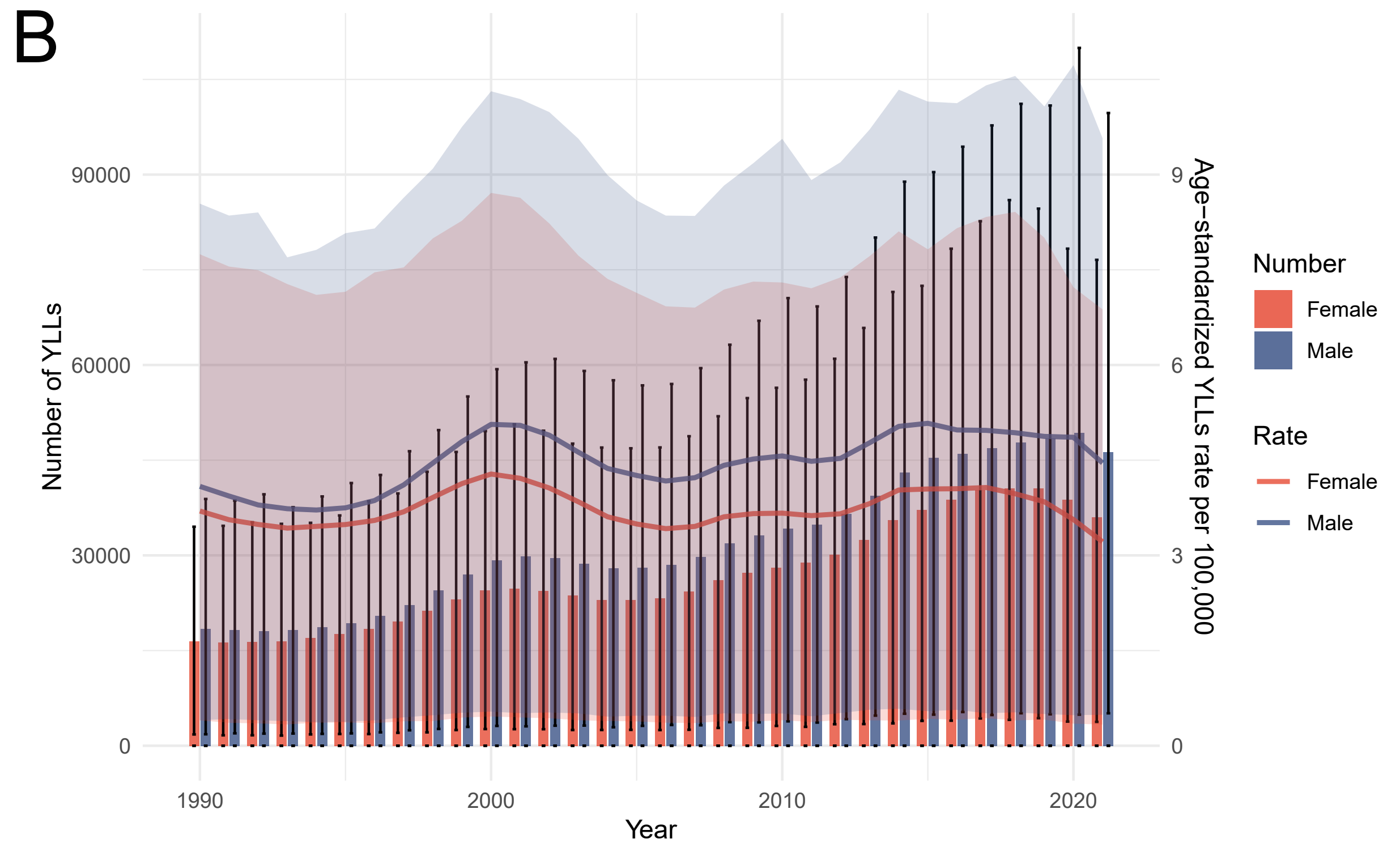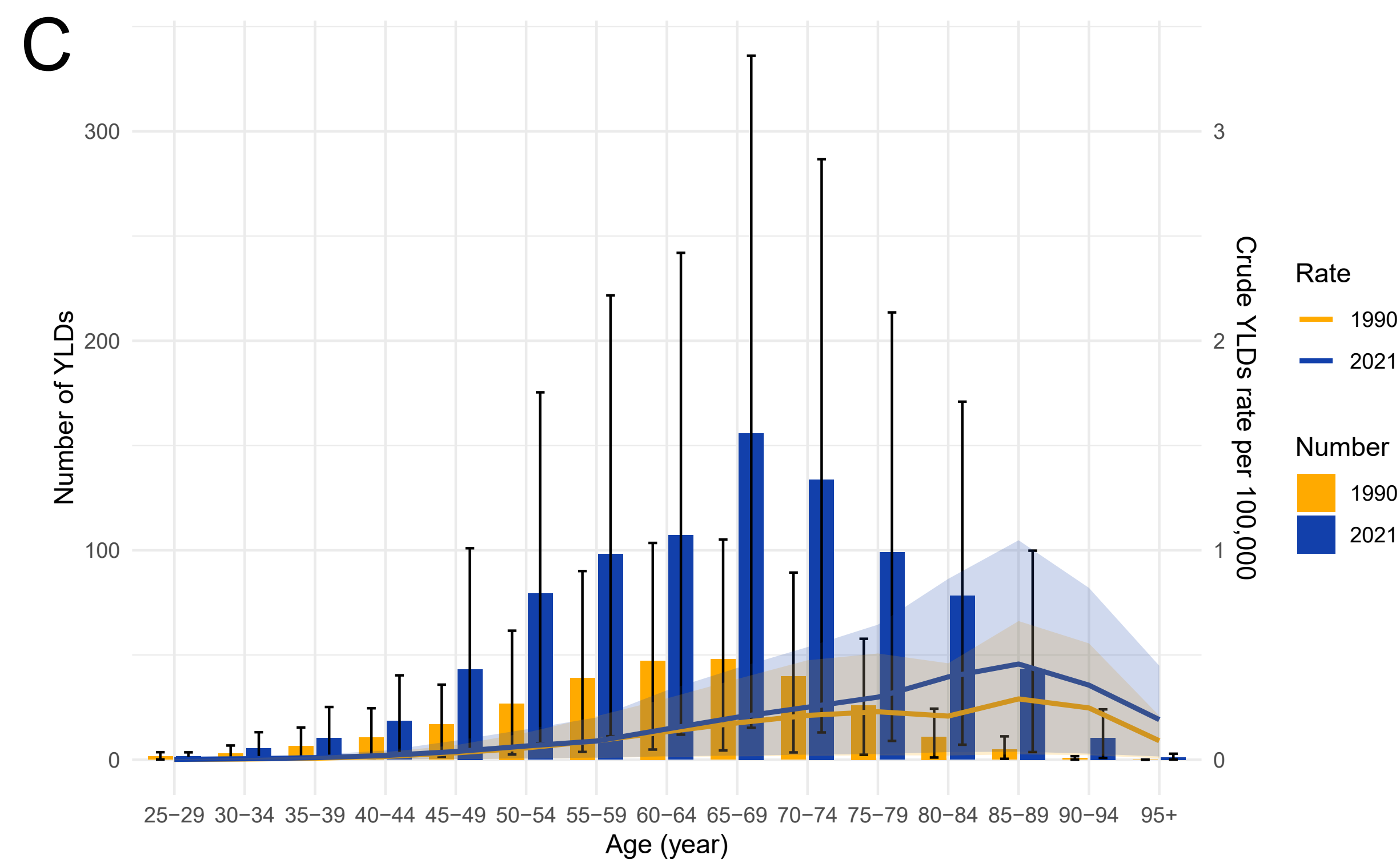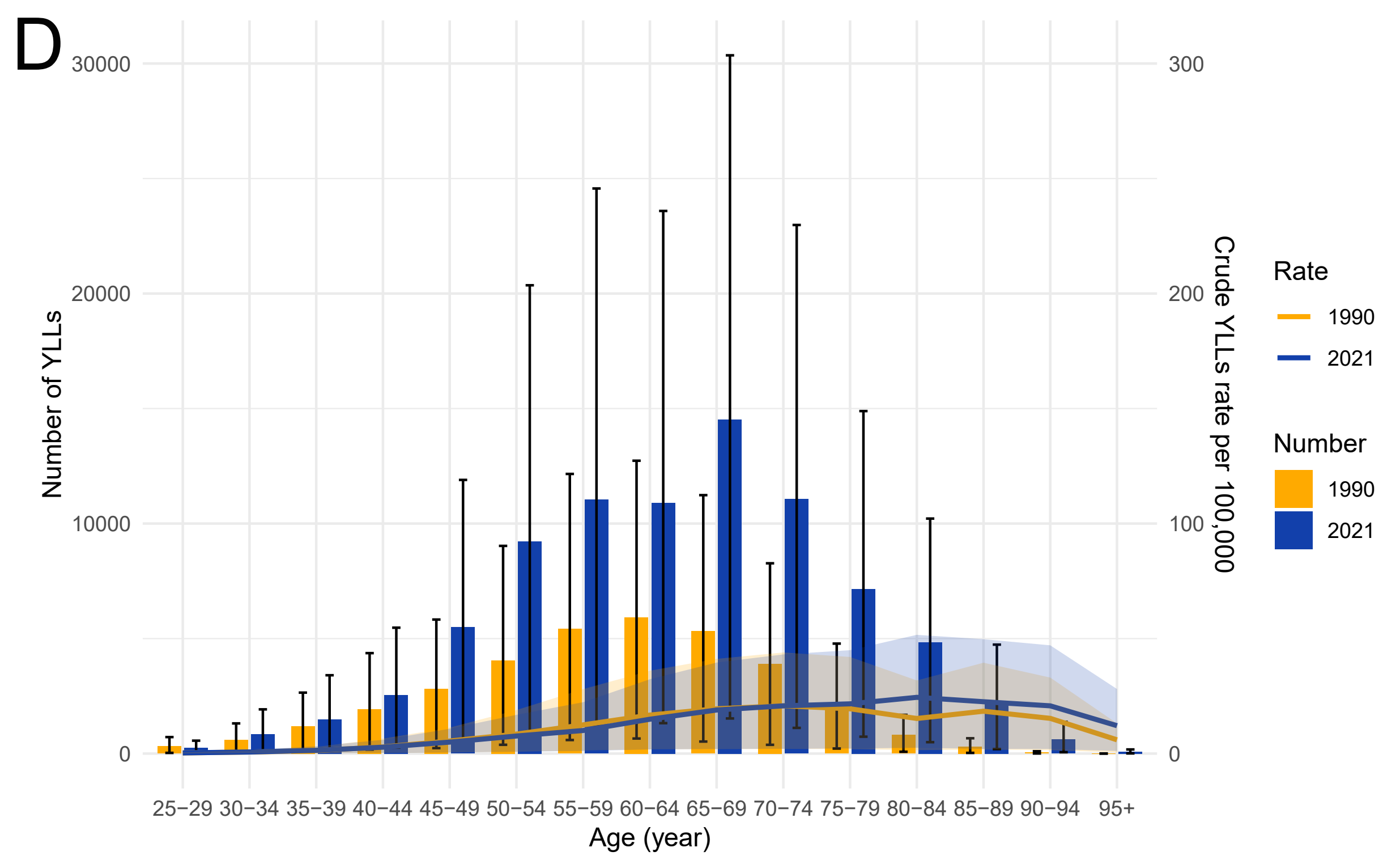

Supplement: SUPPLEMENTARY FIGURE S2 — Temporal trends and age-specific distribution of YLDs and YLLs due to liver cancer attributable to HFPG in China, 1990–2021. (A) Number and age-standardized rates of YLDs by sex over time. (B) Number and age-standardized rates of YLLs by sex over time. (C) Number of YLDs and crude YLD rates per 100,000 by age group in 1990 and 2021. (D) Number of YLLs and crude YLL rates per 100,000 by age group in 1990 and 2021. HFPG, high fasting plasma glucose; YLDs, years lived with disability; YLLs, years of life lost. [file Image_2.pdf]

A

1990–2021 global age-standardized rate

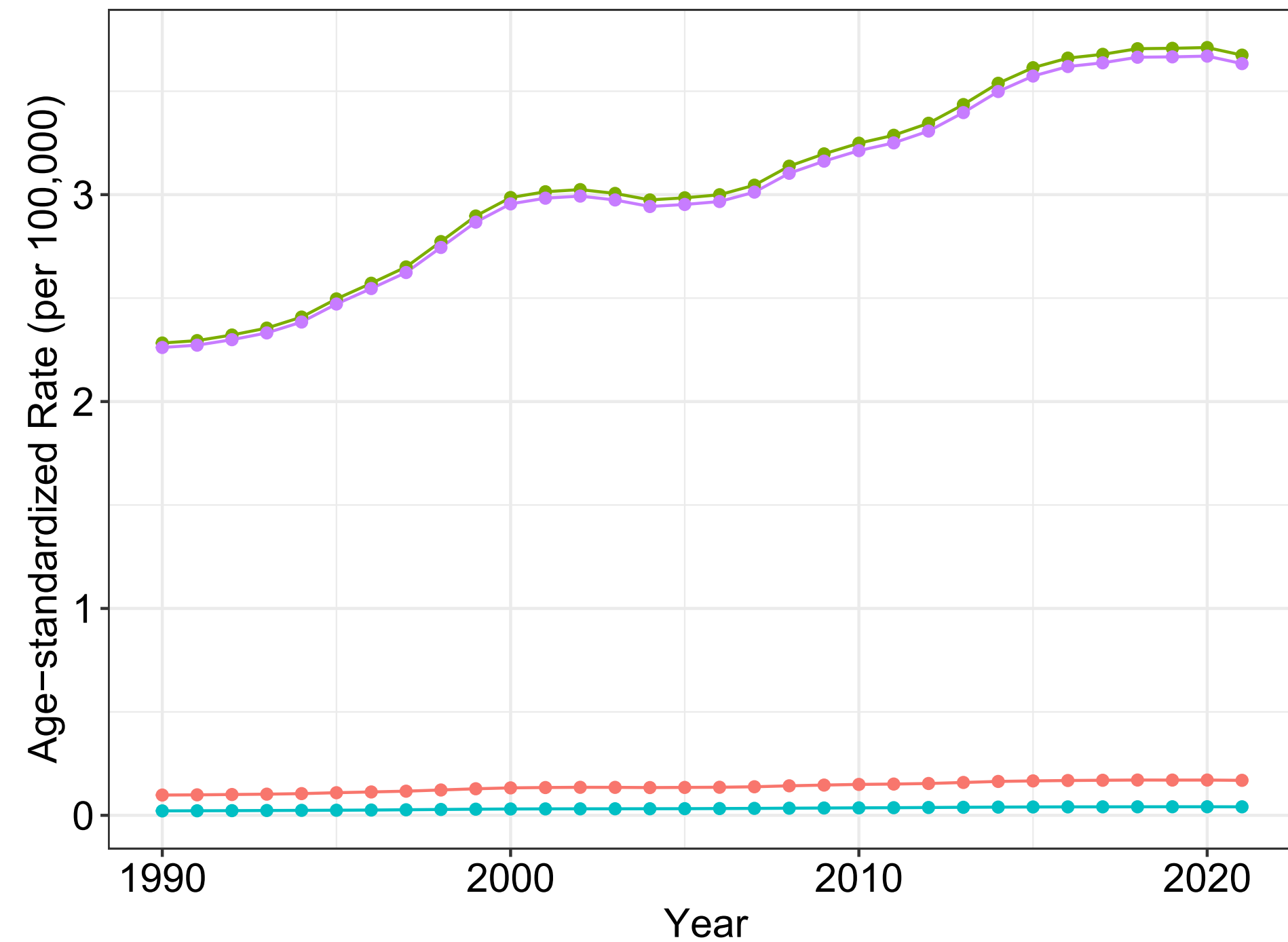

B

1990–2021 China age-standardized rate

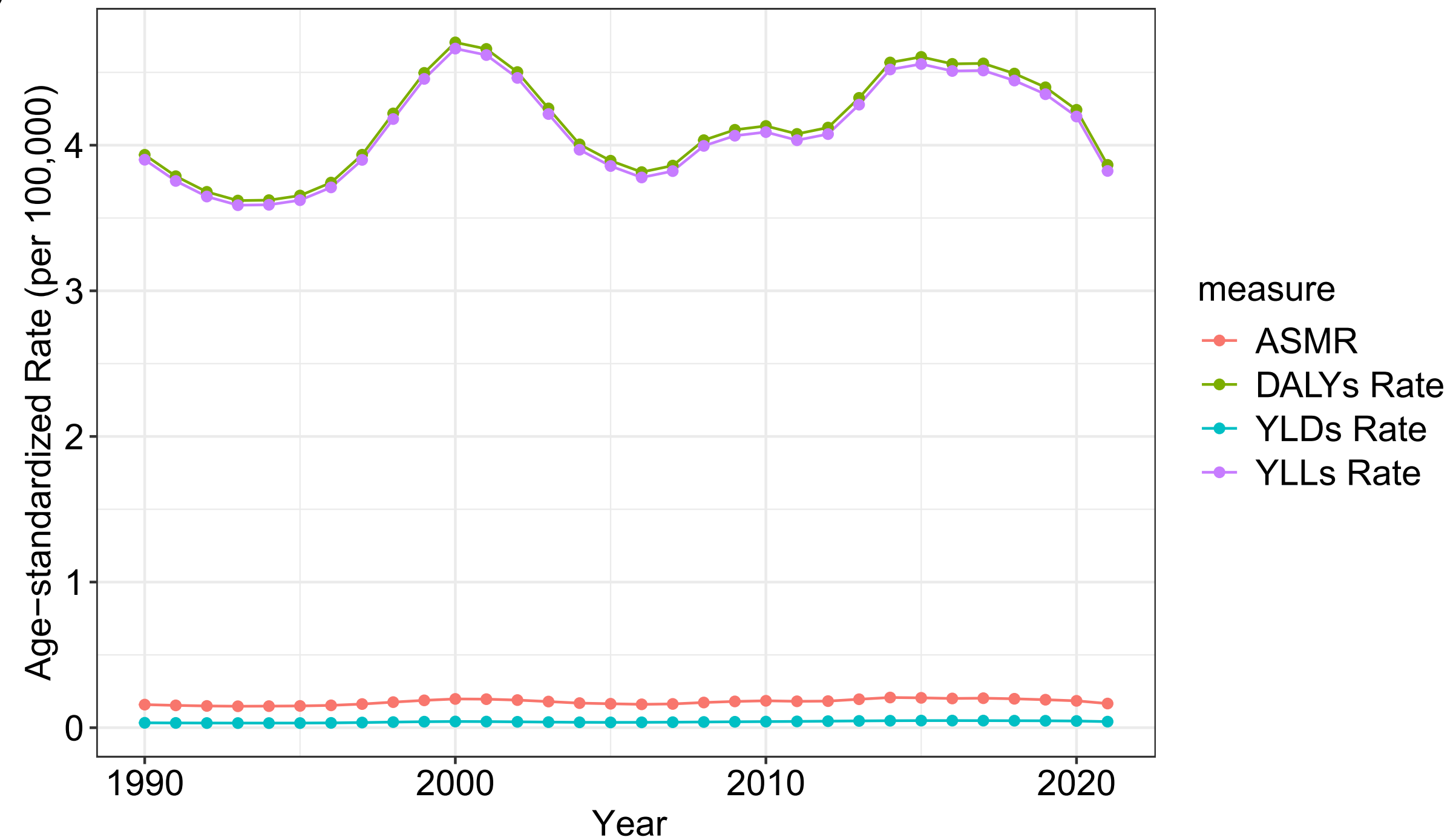

Supplement: SUPPLEMENTARY FIGURE S3 — Trends in global and China-specific age-standardized rates of liver cancer attributable to HFPG from 1990 to 2021. (A) Global trends. (B) Trends in China. The figure illustrates the ASMR, DALYs rate, YLDs rate, and YLLs rate per 100,000 population over time. HFPG, high fasting plasma glucose; ASMR, age-standardized mortality rates; DALYs, disability-adjusted life years; YLDs, years lived with disability; YLLs, years of life lost. [file Image_3.pdf]

A

Multiple Joinpoint Models

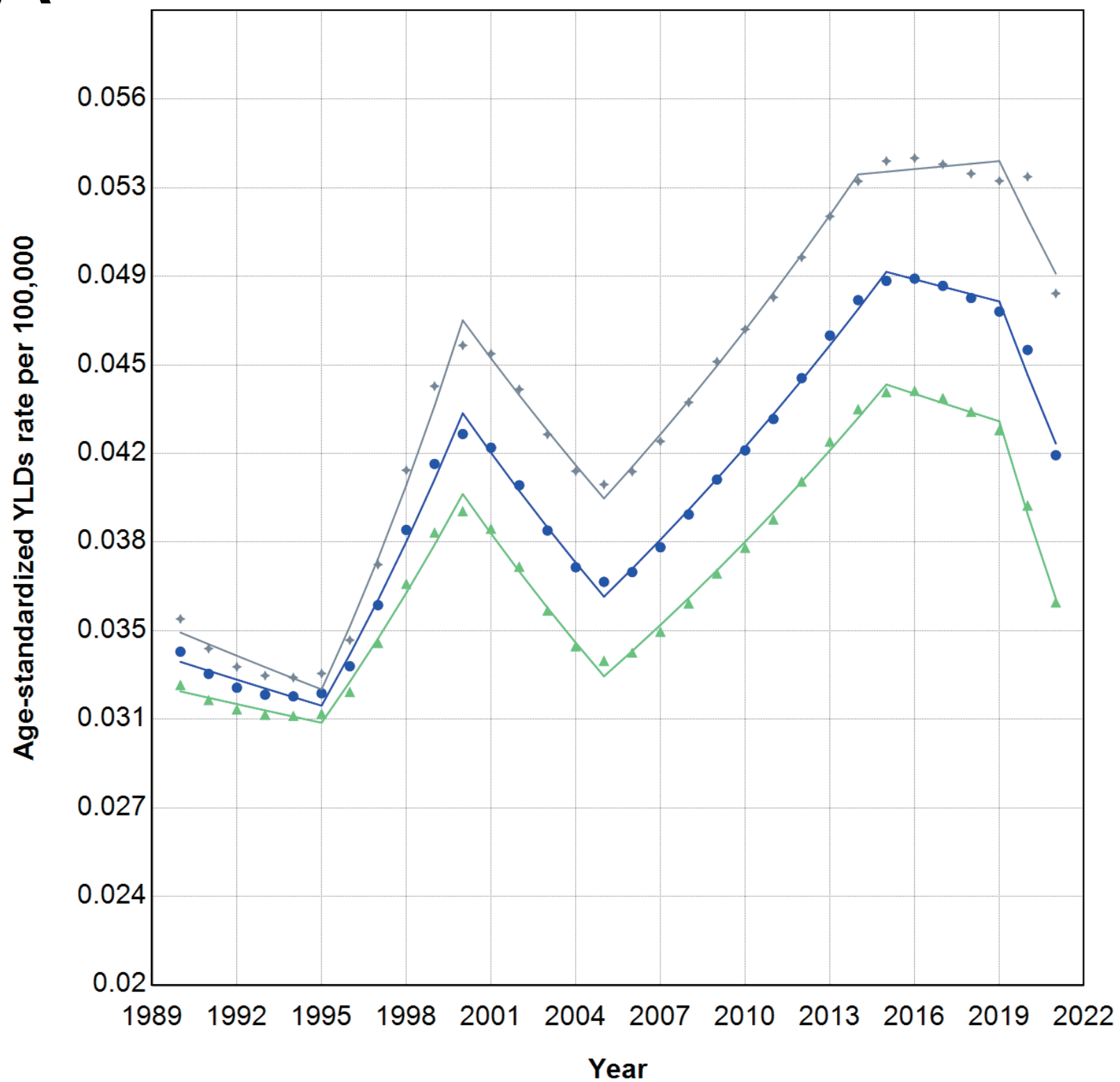

B

Multiple Joinpoint Models

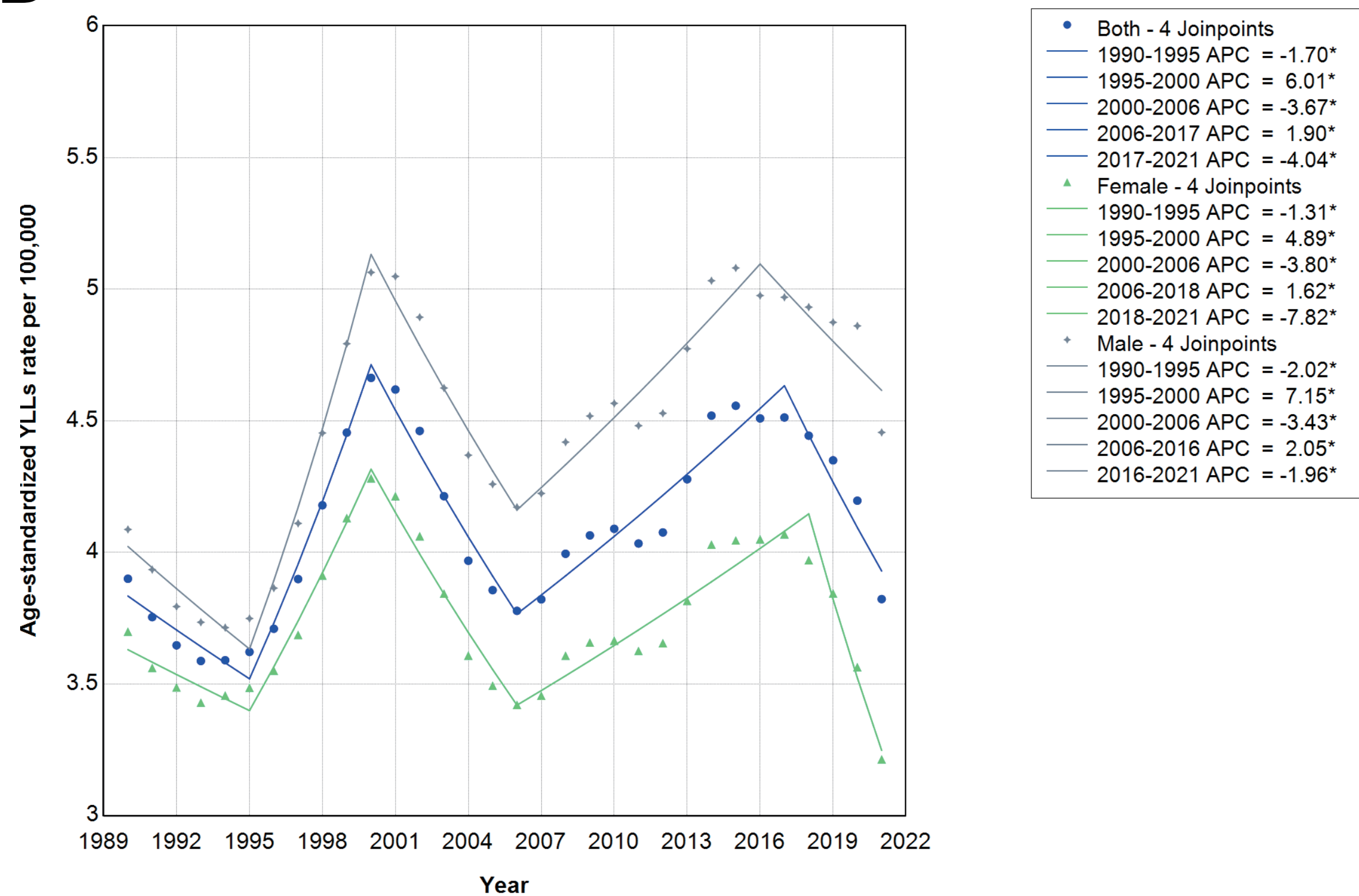

Supplement: SUPPLEMENTARY FIGURE S4 — Joinpoint analysis of trends in age-standardized burden rates of liver cancer attributable to HFPG in China from 1990 to 2021. (A) YLDs rate. (B) YLLs rate. The analysis includes trends for both sexes combined (blue line), females (green line), and males (grey line), with asterisks indicating statistically significant changes (p < 0.05). YLDs, years lived with disability; YLLs, years of life lost; HFPG, high fasting plasma glucose. [file Image_4.pdf]

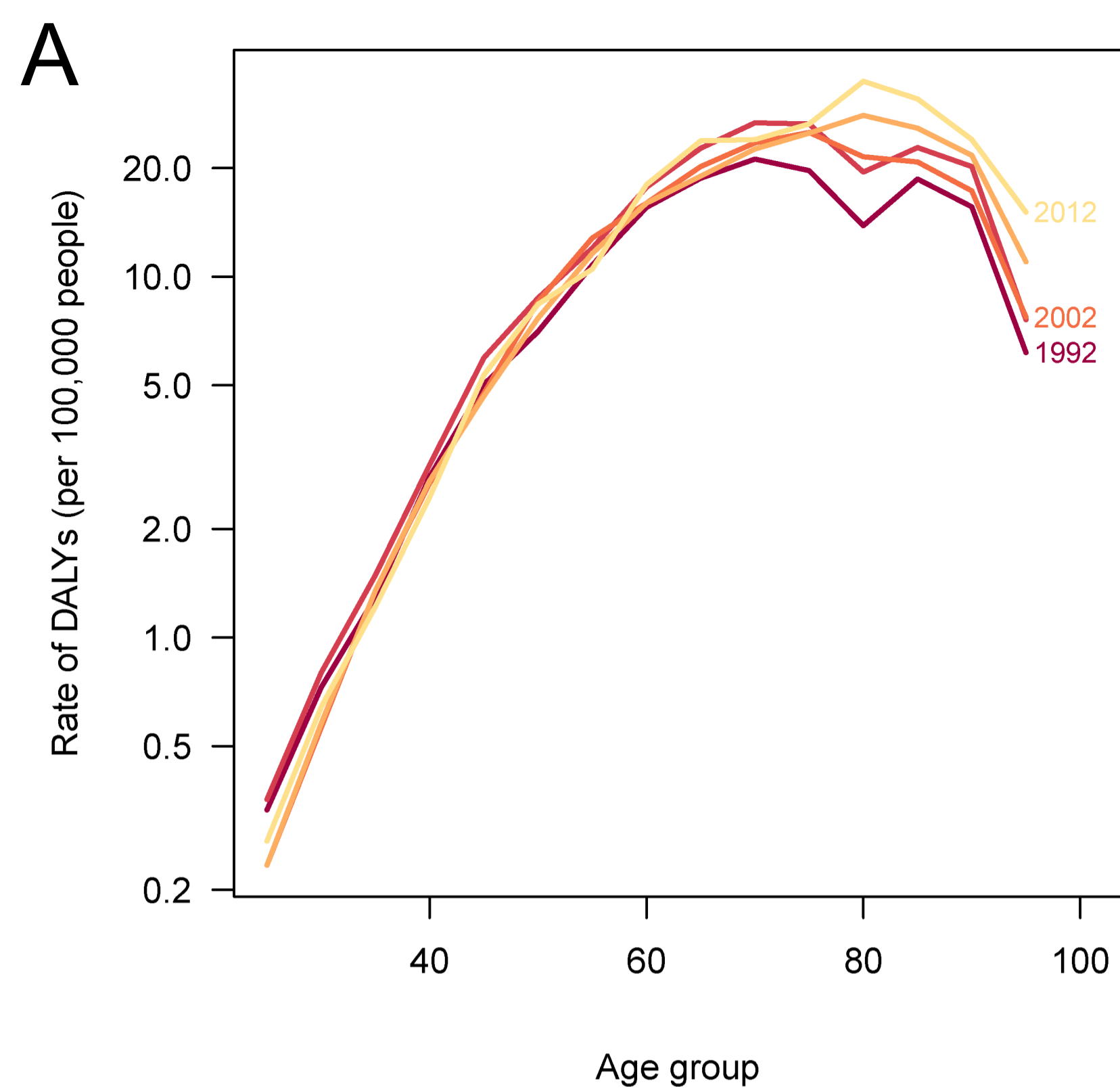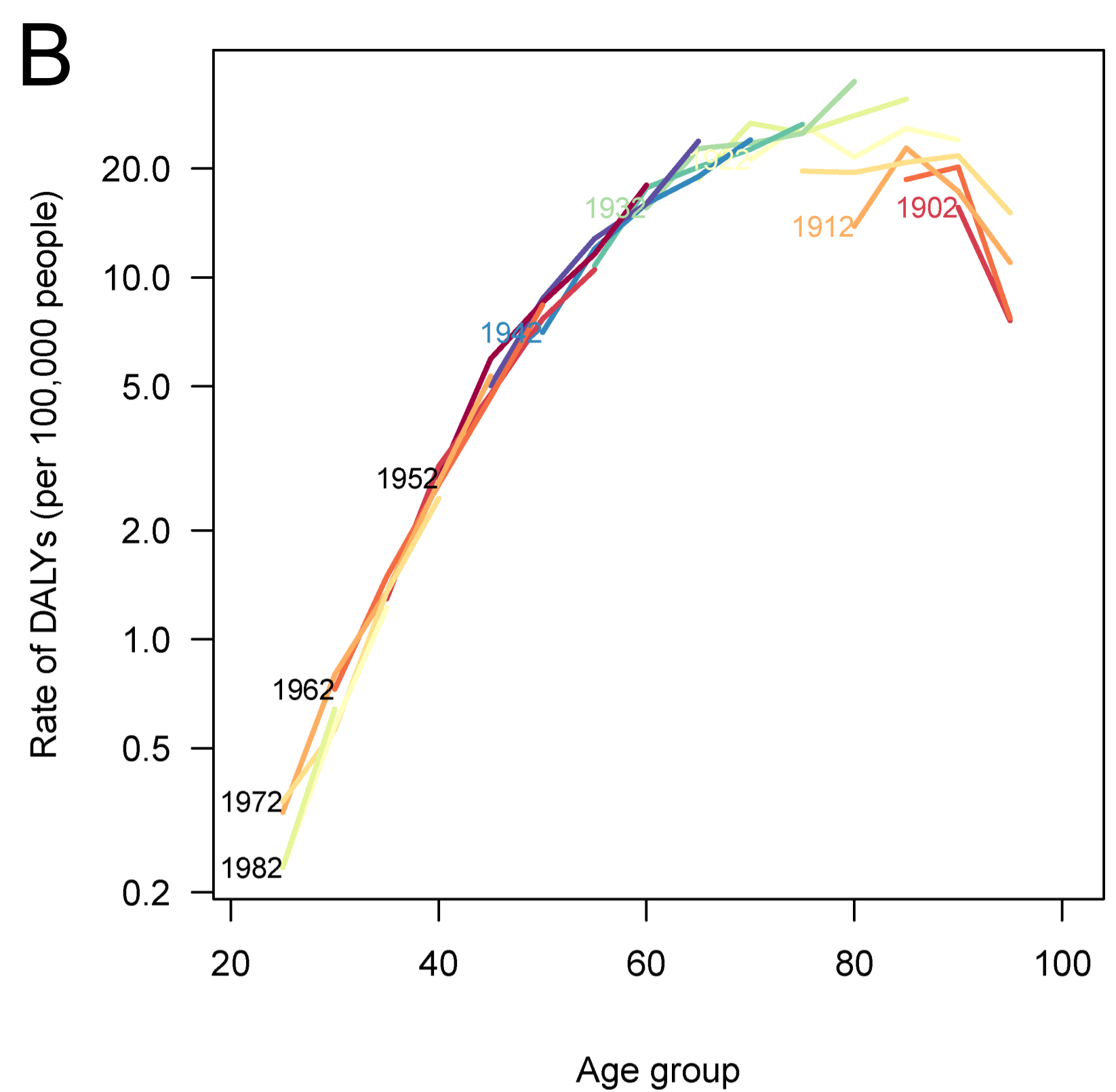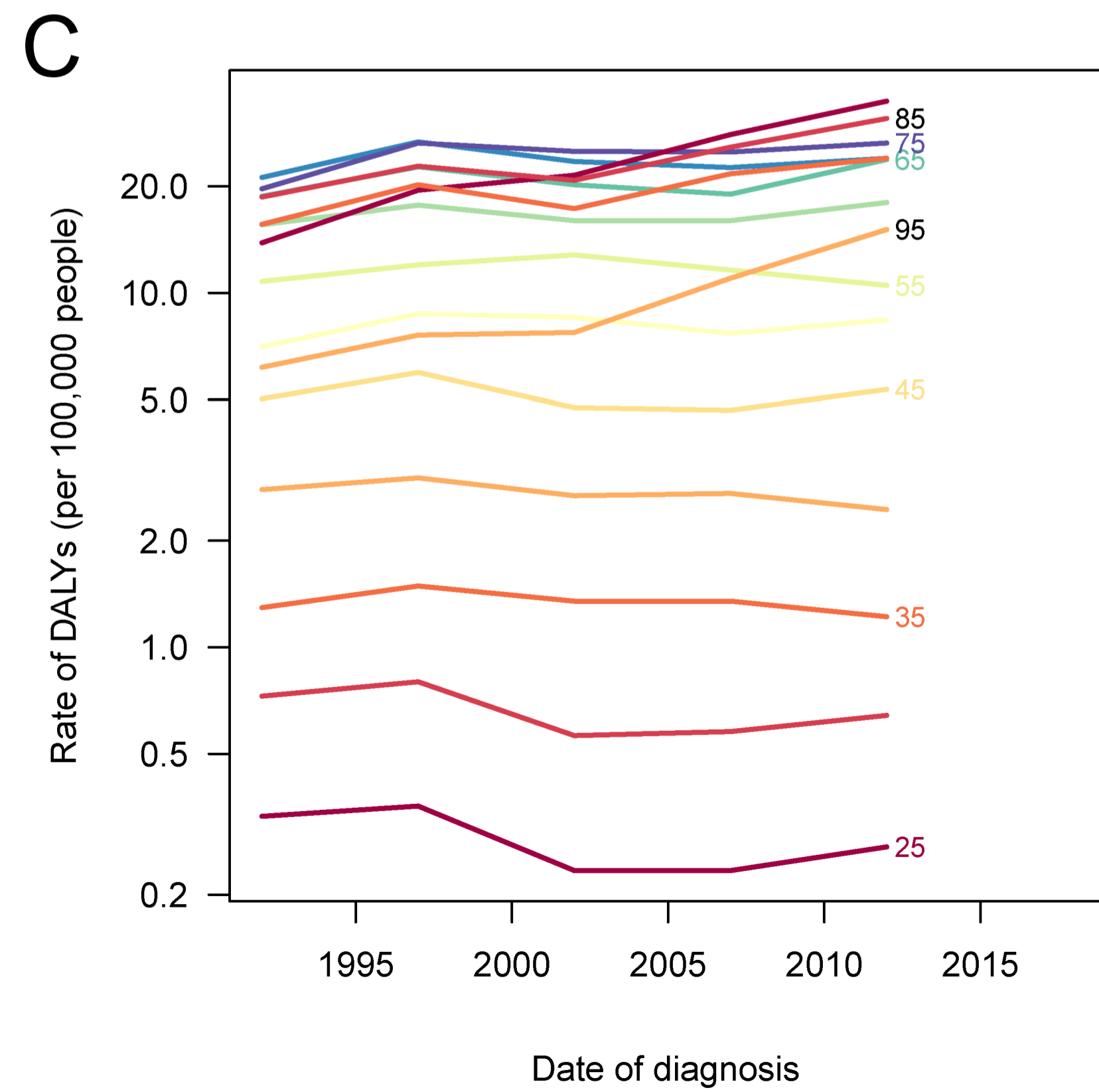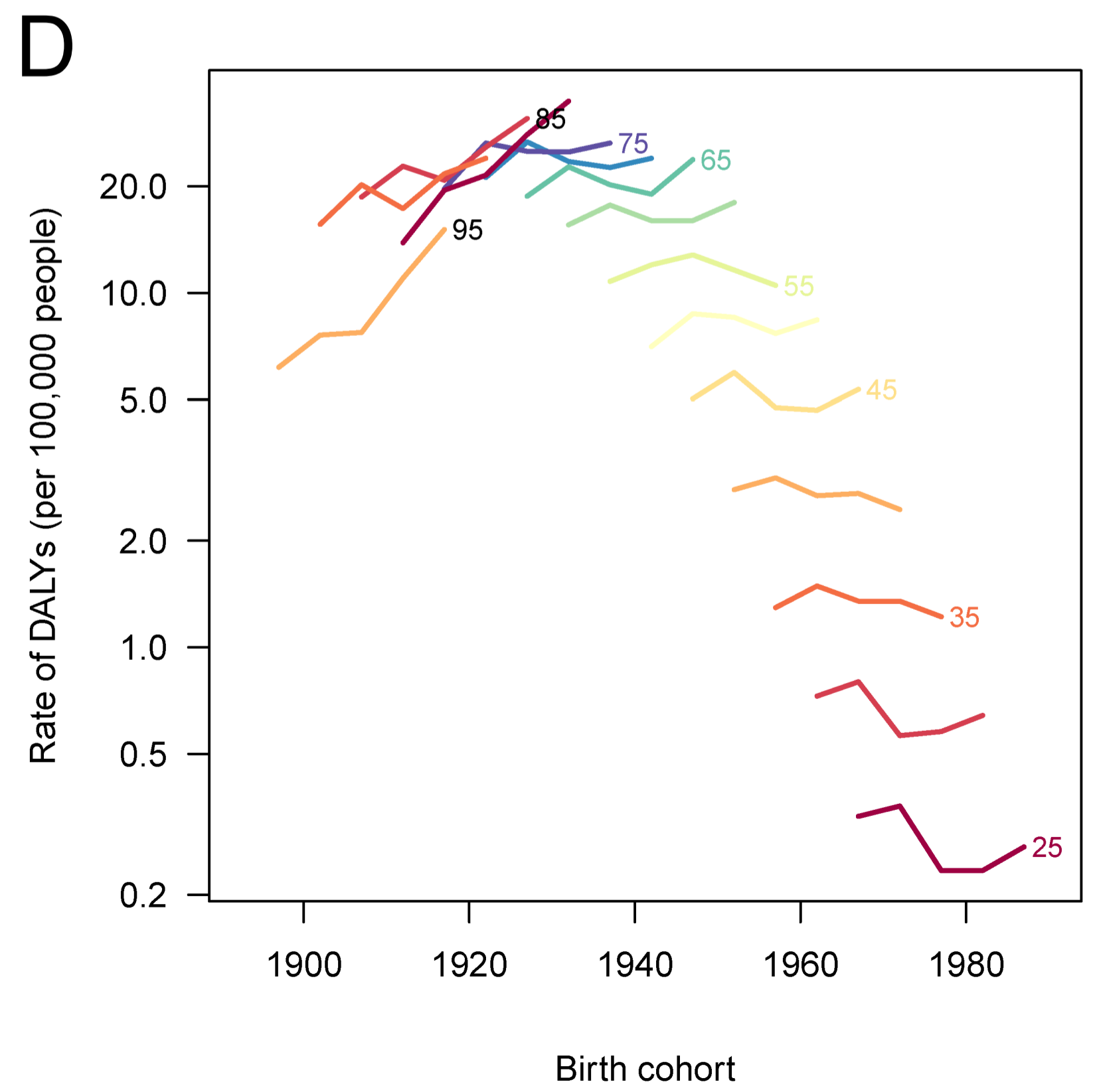

Supplement: SUPPLEMENTARY FIGURE S5 — Age, period, and cohort effects on liver cancer DALYs attributable to HFPG in China. (A) Age-specific DALYs across different time periods, showing how DALYs shift across age groups from 1990 to 2021. (B) Period-specific DALYs across different age groups, illustrating changes in the burden over various time periods. (C) Cohort-specific DALYs, highlighting trends among different birth cohorts, showing how the liver cancer burden has evolved across generations. (D) Period-specific DALY rates across different birth cohorts, illustrating shifts in the burden over time and cohort effects on DALYs associated with HFPG-related liver cancer. HFPG, high fasting plasma glucose; DALYs, disability-adjusted life years. [file Image_5.pdf]
