# Supplementary material for: Impact of high fasting plasma glucose on liver cancer burden in China: a comprehensive analysis of trends from 1990 to 2021
Source: Front Nutr. 2025 Sep 9;12:1628726. doi: 10.3389/fnut.2025.1628726 (PMC12454373; doi:10.3389/fnut.2025.1628726)
Supplement: Supplementary file 6 [file Table_1.DOCX]

Table 3. Joinpoint regression analysis of trends in age-standardized mortality, DALY, YLD, and YLL rates (per 100,000 persons) by sex from 1990 to 2021 for liver cancer attributable to HFPG in China.

|  | Age-standardized mortality rate | | | Age-standardized DALY rate | | | Age-standardized YLD rate | | | Age-standardized YLL rate | | |
| --- | --- | --- | --- | --- | --- | --- | --- | --- | --- | --- | --- | --- |
| Gender | Period | APC (95% CI) | AAPC (95% CI) | Period | APC (95% CI) | AAPC (95% CI) | Period | APC (95% CI) | AAPC (95% CI) | Period | APC (95% CI) | AAPC (95% CI) |
| Both | 1990-1995 | -1.41 (-2.80 - 0.00) | 0.23 (-0.47 - 0.94) | 1990-1995 | -1.69 (-2.95 - -0.42) ^*^ | 0.09 (-0.39 - 0.57) | 1990-1995 | -1.11 (-1.45 - -0.76) ^*^ | 0.77 (0.59 - 0.95) ^*^ | 1990-1995 | -1.70 (-2.96 - -0.42) ^*^ | 0.08 (-0.40 - 0.56) |
|  | 1995-2000 | 6.57 (4.46 - 8.73) ^*^ |  | 1995-2000 | 6.01 (4.12 - 7.94) ^*^ |  | 1995-2000 | 6.67 (6.15 - 7.19) ^*^ |  | 1995-2000 | 6.01 (4.10 - 7.96) ^*^ |  |
|  | 2000-2006 | -3.55 (-4.87 - -2.21) ^*^ |  | 2000-2006 | -3.66 (-4.86 - -2.44) ^*^ |  | 2000-2005 | -3.73 (-4.20 - -3.25) ^*^ |  | 2000-2006 | -3.67 (-4.88 - -2.44) ^*^ |  |
|  | 2006-2015 | 2.83 (2.15 - 3.51) ^*^ |  | 2006-2017 | 1.91 (1.48 - 2.35) ^*^ |  | 2005-2015 | 3.20 (3.06 - 3.35) ^*^ |  | 2006-2017 | 1.90 (1.46 - 2.34) ^*^ |  |
|  | 2015-2019 | -1.47 (-4.46 - 1.62) |  | 2017-2021 | -4.04 (-5.79 - -2.26) ^*^ |  | 2015-2019 | -0.62 (-1.36 - 0.12) |  | 2017-2021 | -4.04 (-5.80 - -2.24) ^*^ |  |
|  | 2019-2021 | -7.24 (-12.93 - -1.18) ^*^ |  |  |  |  | 2019-2021 | -6.26 (-7.70 - -4.80) ^*^ |  |  |  |  |
| Female | 1990-1995 | -1.04 (-2.44 - 0.39) | -0.09 (-0.80 - 0.63) | 1990-1995 | -1.30 (-2.42 - -0.17) ^*^ | -0.35 (-0.79 - 0.09) | 1990-1995 | -0.82 (-1.13 - -0.50) ^*^ | 0.36 (0.20 - 0.53) ^*^ | 1990-1995 | -1.31 (-2.44 - -0.17) ^*^ | -0.36 (-0.80 - 0.09) |
|  | 1995-2000 | 5.40 (3.32 - 7.52) ^*^ |  | 1995-2000 | 4.89 (3.24 - 6.57) ^*^ |  | 1995-2000 | 5.46 (4.99 - 5.94) ^*^ |  | 1995-2000 | 4.89 (3.22 - 6.59) ^*^ |  |
|  | 2000-2006 | -3.81 (-5.15 - -2.46) ^*^ |  | 2000-2006 | -3.79 (-4.86 - -2.71) ^*^ |  | 2000-2005 | -4.03 (-4.46 - -3.60) ^*^ |  | 2000-2006 | -3.80 (-4.88 - -2.71) ^*^ |  |
|  | 2006-2015 | 3.00 (2.32 - 3.69) ^*^ |  | 2006-2018 | 1.63 (1.29 - 1.97) ^*^ |  | 2005-2015 | 3.17 (3.04 - 3.30) ^*^ |  | 2006-2018 | 1.62 (1.28 - 1.96) ^*^ |  |
|  | 2015-2019 | -1.58 (-4.62 - 1.55) |  | 2018-2021 | -7.82 (-10.18 - -5.41) ^*^ |  | 2015-2019 | -0.86 (-1.55 - -0.16) ^*^ |  | 2018-2021 | -7.82 (-10.19 - -5.38) ^*^ |  |
|  | 2019-2021 | -9.87 (-15.47 - -3.91) ^*^ |  |  |  |  | 2019-2021 | -8.81 (-10.11 - -7.50) ^*^ |  |  |  |  |
| Male | 1990-1995 | -1.76 (-3.41 - -0.08) ^*^ | 0.66 (0.05 - 1.29) ^*^ | 1990-1995 | -2.01 (-3.34 - -0.66) ^*^ | 0.45 (-0.05 - 0.95) | 1990-1995 | -1.39 (-1.81 - -0.98) ^*^ | 1.15 (0.94 - 1.37) ^*^ | 1990-1995 | -2.02 (-3.35 - -0.66) ^*^ | 0.44 (-0.06 - 0.95) |
|  | 1995-2000 | 8.03 (5.48 - 10.64) ^*^ |  | 1995-2000 | 7.16 (5.14 - 9.22) ^*^ |  | 1995-2000 | 8.02 (7.36 - 8.69) ^*^ |  | 1995-2000 | 7.15 (5.12 - 9.23) ^*^ |  |
|  | 2000-2006 | -3.12 (-4.69 - -1.53) ^*^ |  | 2000-2006 | -3.42 (-4.68 - -2.15) ^*^ |  | 2000-2005 | -3.30 (-3.90 - -2.70) ^*^ |  | 2000-2006 | -3.43 (-4.70 - -2.15) ^*^ |  |
|  | 2006-2015 | 2.58 (1.77 - 3.39) ^*^ |  | 2006-2016 | 2.06 (1.52 - 2.60) ^*^ |  | 2005-2014 | 3.24 (3.03 - 3.45) ^*^ |  | 2006-2016 | 2.05 (1.50 - 2.59) ^*^ |  |
|  | 2015-2021 | -2.16 (-3.39 - -0.91) ^*^ |  | 2016-2021 | -1.96 (-3.31 - -0.59) ^*^ |  | 2014-2019 | 0.20 (-0.38 - 0.79) |  | 2016-2021 | -1.96 (-3.32 - -0.58) ^*^ |  |
|  |  |  |  |  |  |  | 2019-2021 | -4.39 (-6.26 - -2.48) ^*^ |  |  |  |  |

Abbreviations: AAPC, average annual percent change presented for full period; APC, annual percent change; HFPG, high fasting plasma glucose; CI, confidence interval; ^*^, *p* <0.05 (permutation test).
